# Supplemental Donor Milk vs Infant Formula in Moderate to Late Preterm Infants: A Randomized Clinical Trial

**DOI:** 10.1001/jamapediatrics.2025.2365

**Published:** 2025-08-04

**Authors:** Alice R. Rumbold, Melissa M. Lai, Deanne August, Pieter Koorts, Tim Donovan, Lisa Yelland, Maria Makrides, Alana R. Cuthbert, Laura D. Klein, Teresa Ginis, Aya Al Gharram, Susie Jones, Laura Summers, Andrew McPhee, Amy Keir

**Affiliations:** 1 Women and Kids Theme, South Australian Health and Medical Research Institute, Adelaide, South Australia, Australia; 2 Women and Babies Division, Women’s and Children’s Health Network, Adelaide, South Australia, Australia; 3 Adelaide Medical School, The University of Adelaide, Adelaide, South Australia, Australia; 4 Royal Brisbane and Women’s Hospital, Brisbane, Queensland, Australia; 5 School of Medicine, University of Queensland, St Lucia, Queensland, Australia; 6 School of Public Health, The University of Adelaide, Adelaide, South Australia, Australia; 7 Australian Red Cross Lifeblood, Sydney, New South Wales, Australia

## Abstract

**Importance:**

High-quality evidence supports the use of pasteurized donor human milk (donor milk) in very preterm infants with insufficient maternal milk available. However, evidence to guide the use of donor milk in more mature preterm infants is lacking.

**Objective:**

To compare the effect of donor milk vs term infant formula, used to supplement insufficient maternal milk, on the time to establish full enteral feeds in moderate to late preterm infants.

**Design, Setting, and Participants:**

This multisite, blinded randomized clinical trial was conducted from July 6, 2021, to April 5, 2023, at 2 Australian neonatal units. Infants 4 days old or younger, born between 32 + 0 and 36 + 6 weeks’ gestation, with a birth weight of 1500 g or higher, and admitted to a neonatal unit were eligible if they were clinically stable, ready to commence or had commenced enteral feeds, and had insufficient maternal milk available. Infants were followed up until 6-month corrected age (CA). Follow-up assessments until 6-month CA were completed by December 4, 2023, and data analyses were completed by January 23, 2025.

**Intervention:**

Infants were randomly assigned to receive supplemental donor milk or term formula for up to 8 days, stratified by site and gestational age at birth.

**Main Outcomes and Measures:**

The primary outcome was time to full enteral feeds (defined as 150 mL/kg/day). Secondary outcomes included feed intolerance, growth, body composition, breast milk feeding, and hospital readmissions to 6-month CA.

**Results:**

Of 201 infants randomized (99 to donor milk, 102 to formula), the mean (SD) birth gestational age was 34.6 (1.2) weeks, mean (SD) birth weight was 2267.1 (450.8) g, 88 infants (43.8%) were female, and 75 infants (37.3%) were a twin or triplet. Mean (SD) time to reach full enteral feeds did not differ between groups (donor milk group: 5.7 [2.6] days; formula group: 5.8 [3.4] days; adjusted mean difference, −0.07; 95% CI, −0.90 to 0.76). Secondary outcomes were similar between groups, except that infants in the donor milk group had a lower rate of birth weight regain compared with the formula group (mean [SD] time to regain in donor milk group: 10.7 [5.7] days; formula group: 8.4 [4.4] days; hazard ratio, 0.65; 95% CI, 0.47-0.88).

**Conclusions and Relevance:**

In this multisite randomized clinical trial, supplemental donor milk did not reduce time to full enteral feeds in moderate to late preterm infants compared with term formula for up to 8 days.

**Trial Registration:**

anzctr.org.au Identifier: ACTRN12621000529842

## Key Points

**Question:**

What is the effect of providing pasteurized donor human milk (donor milk) compared with term infant formula, both used to supplement insufficient maternal milk, on the time to establish full enteral feeds in moderate to late preterm infants?

**Findings:**

In this blinded randomized clinical trial that included 201 infants, the time to reach full enteral feeds was not significantly different between infants given supplemental donor milk compared with term formula for up to 8 days.

**Meaning:**

In moderate to late preterm infants with insufficient maternal milk available, the type of milk supplement used during the neonatal hospitalization did not affect their feeding progression.

## Teaser

This randomized clinical trial conducted in 2 Australian neonatal units compares the effect of donor milk vs term infant formula on the time to establish full enteral feeds in moderate to late preterm infants.

## Introduction

Infants born between 32 and 36 weeks’ gestation (moderate to late preterm) comprise approximately 85% of all preterm births.^1^ While the vast majority survive, many need specialized neonatal care for respiratory, thermoregulation, and/or feeding support.^2,3^ In the longer term, children born moderate to late preterm have increased risks of cognitive and motor impairment,^4,5,6^ behavioral difficulties,^6,7^ and cardiometabolic diseases^8,9^ compared to children born full term.

Maternal milk is the optimal source of nutrition for all infants.^10,11^ Moderate to late preterm infants have more difficulties establishing breastfeeding than full-term infants. Preterm birth can delay milk production,^12^ and the infant’s gastrointestinal immaturity may necessitate tube feeding initially, with a gradual transition to breastfeeding.^10,13,14^ Supplemental nutrition is usually required until there is sufficient maternal milk intake to meet their high nutrient needs and avoid hypoglycemia and poor growth.^10^ However, there is no clear guidance about the optimal management of nutrition support in this population,^10^ and practices vary widely.^15^

In very preterm infants, randomized clinical trials demonstrate that supplemental pasteurized donor human milk (donor milk) is associated with a reduced risk of necrotizing enterocolitis (NEC) compared with formula; however, weight gain may be slower.^16^ NEC is rare in moderate to late preterm infants—nevertheless, there is increasing interest in the use of donor milk in this population.^17,18^ While not equivalent to maternal milk, donor milk retains many of the bioactive components unique to human milk.^19,20^ Further, feed tolerance is improved with donor milk compared with formula^16,21^; therefore, donor milk may promote a faster progression to full milk feeds and breastfeeding. There is limited evidence to guide the use of donor milk in moderate to late preterm infants, as trials examining impacts in this population are lacking.^10,22^ This randomized clinical trial aimed to compare the effect of donor milk vs term formula, used to supplement insufficient maternal milk, on the time to establish full enteral feeds in moderate to late preterm infants.

## Methods

### Study Design

The trial was a multisite, blinded, parallel-group, partially clustered randomized clinical trial conducted at 2 Australian neonatal units: the Women’s and Children’s Hospital in South Australia and the Royal Brisbane and Women’s Hospital (RBWH) in Queensland. Infants were enrolled from July 6, 2021, to April 5, 2023, with follow-up assessments until 6-month corrected age (completed by December 4, 2023). The trial protocol (see Supplement 1) was approved by the Women’s and Children’s Health Network Human Research Ethics Committee in South Australia (HREC/20/WCHN/126), with governance approval granted at each site. The results are reported in accordance with the Consolidated Standards of Reporting Trials (CONSORT) reporting guideline.

### Participants

Infants were eligible if they met all of the following criteria: born between 32 + 0 and 36 + 6 completed weeks’ gestation; birth weight of 1500 g or higher; admitted to the neonatal unit at a participating site; clinically stable as determined by the treating health care team; ready to commence enteral feeds or commenced enteral feeds but insufficient maternal milk available; aged 4 days old or younger; and had a parent or guardian capable of giving informed consent. Only infants with a gestation of 34 + 0 weeks or more at birth were enrolled at RBWH, as the provision of donor milk to infants born less than 34 weeks was standard practice at this site.

Infants with any of the following criteria were excluded: (1) major congenital malformation either likely to interfere with the ability to ingest milk or requiring surgery in the first 6 months of life; (2) metabolic disorder that precludes breastfeeding; or (3) had received formula before randomization.

After written consent was obtained from the parent or guardian, infants were randomly allocated to either the intervention or control group in a 1:1 ratio using a secure web-based randomization service (REDCap [Vanderbilt]).^23^ Stratification occurred for site and gestational age (GA) at birth (<34 weeks, ≥34 weeks). A computer-generated randomization schedule using a balanced variable block design was prepared and held by an independent statistician not otherwise involved in the trial. Singletons were randomized independently, while twins and triplets were cluster randomized to the same treatment arm. Infants were randomized when the amount of maternal milk available was deemed insufficient to meet the infant’s enteral feed requirements.

### Intervention

Following randomization, infants received the intervention or control group supplementary milk for up to 8 days. The supplementary milk was ceased prior to 8 days if there was sufficient maternal milk to meet the target fluid intake or if the infant was transferred to the postnatal ward or another hospital or discharged home.

The intervention group received donor milk provided by Australian Red Cross Lifeblood. Analysis of 200 donor milk batches found the mean (range) crude protein content of donor milk was 1.16 g/100 mL (0.7- to 1.96), and mean (range) fat content was 3.85 g/100 mL (1.46-9.39).^24^ Typical protein and fat concentrations for standard term formula are 1.5 g/100 mL and 3.5 g/100 mL, respectively.^25^ The control group received term formula as per each site’s standard practice. Each site had the option of commencing nutrient fortification during the intervention period by requesting “extra calories” on order forms. Where this occurred, available maternal milk was fortified with bovine human milk fortifier; the intervention group received donor milk fortified with bovine human milk fortifier and the control group received preterm formula.

Participants, families, clinical teams, research nurses, outcome assessors, and data analysts were blinded to the treatment group. To maintain blinding, staff not involved in clinical care, recruitment, or participant follow-up prepared a 24-hour supply of feeds daily in workstations away from clinical bed space. Syringes and bottles containing study milk (donor milk or formula) were covered in yellow tape to mask the appearance. Extra empty covered syringes and bottles of study milk with a lid adaptor for milk aspiration allowed for the decanting of milk if feed volumes were adjusted during those 24 hours. Feeds were administered by nursing and midwifery staff, who were instructed to feed with available maternal milk first.

During the intervention period, the use of formulas not part of the study milk was not permitted. Medical records were monitored to assess adherence. When the intervention period ceased (8 days), if the infant still required supplementary milk, they were fed according to standard practice at each site, which did not include donor milk.

### Data Collection

Baseline demographic and clinical characteristics of infants and mothers were collected from medical records. Staff recorded study milk and maternal milk intake separately in the medical record. This information and the number of direct breastfeeds was examined to calculate the date that full enteral feeds (150 mg/kg/day) were reached. Documented episodes of feed intolerance and use of intravenous (IV) nutrition and other supplements were collected. Additional data obtained from medical records included weight, length, and head circumference at randomization and within 24 hours of intervention completion; neonatal complications; length of hospital stay; and type of milk feeding on discharge.

At discharge and when the infant was 2- and 4-month CA, infant weight, length, and head circumference were measured and body composition was assessed using air displacement plethysmography (COSMED).^26^ Parent-completed questionnaires assessed type of milk feeding and infant hospital readmissions at 2-, 4-, and 6-month CA.

### Outcomes

The primary outcome was the time to reach full enteral feeds (days since randomization), defined as 150 mL/kg/day.

Secondary outcomes included the following factors: frequency of feed intolerance, defined as the number of episodes after randomization where there was a medical decision to stop or reduce oral feeds (during the intervention period and separately until discharge); time to establish full suck feeds; duration of use of IV glucose; and duration of use of parenteral nutrition.

Other secondary outcomes included the following factors: time to regain birth weight (in days); weight *z* score for gestational age and change in weight *z* score from birth to initial discharge home using Fenton growth charts^27^; weight, length, and head circumference at discharge, 2-, and 4-month CA; confirmed late-onset sepsis; confirmed NEC (Bell stage 2 or more); length of initial hospital stay, frequency, and length of any readmissions up to 6-month CA; body composition, including fat-free mass as a percentage of total body mass at discharge, 2-, and 4-month CA; and breast milk feeding (exclusive or any) and method of feeding (via bottle, at breast) at discharge, 2-, 4-, and 6-month CA.

### Sample Size and Statistical Analysis

A sample size of 100 infants per group provides more than 80% power to detect a reduction in the mean time to full enteral feeds (in days) between the treatment groups of 0.5 standard deviations (approximately 1.5 days for this trial), with a 2-sided α of 0.05 and 5% loss to follow-up. This calculation assumed that 24% of infants will be twins and allowed for a worst-case scenario of perfect correlation between outcomes of twins. As successful feeding is a key discharge criterion, the expected effect size could translate to a meaningful reduction in length of hospitalization.

Infants were analyzed according to their randomized treatment group, regardless of adherence or the amount of supplementary feeding required. The primary outcome was analyzed using a linear regression model with adjustment for site, GA, and multiple birth to estimate a difference in means. Generalized estimating equations (GEEs) with an independence working correlation structure and robust variance estimation were used to account for partial clustering due to multiple births. Post hoc sensitivity analyses for the primary outcome included adjusting for gestational diabetes, excluding ineligible infants, and restricting to the 34 or more weeks’ GA stratum. Secondary outcomes were analyzed using linear GEEs (continuous outcomes), log binomial GEEs (binary outcomes), negative binomial GEEs (count outcomes), or Cox proportional hazards models with a cluster-adjusted variance estimator and adjustment for site, GA, and multiple birth. Blinding was assessed by treatment group based on parental guesses regarding the assigned group using the blinding index, with values between −0.2 and 0.2 suggesting successful blinding.^28^ Analyses were performed using R version 4.3.2 (R Foundation) and Stata version 18.0 (StataCorp) following a prespecified statistical analysis plan (Supplement 2).

## Results

### Baseline Characteristics

Of 381 infants identified as eligible, 201 (52.8%) were enrolled and randomized: 99 to the donor milk group and 102 to the formula group (82 mothers per group; Figure). The mean (SD) GA at birth was 34.6 (1.2) weeks, mean (SD) birth weight was 2267.1 (450.8) g, 75 infants (37.3%) were a twin or triplet, and 88 (43.8%) were female. The mean (SD) maternal age was 31.7 (5.0) years, and 100 mothers (61.0%) had no prior births. The baseline characteristics were similar between groups (Table 1).

**Figure.**
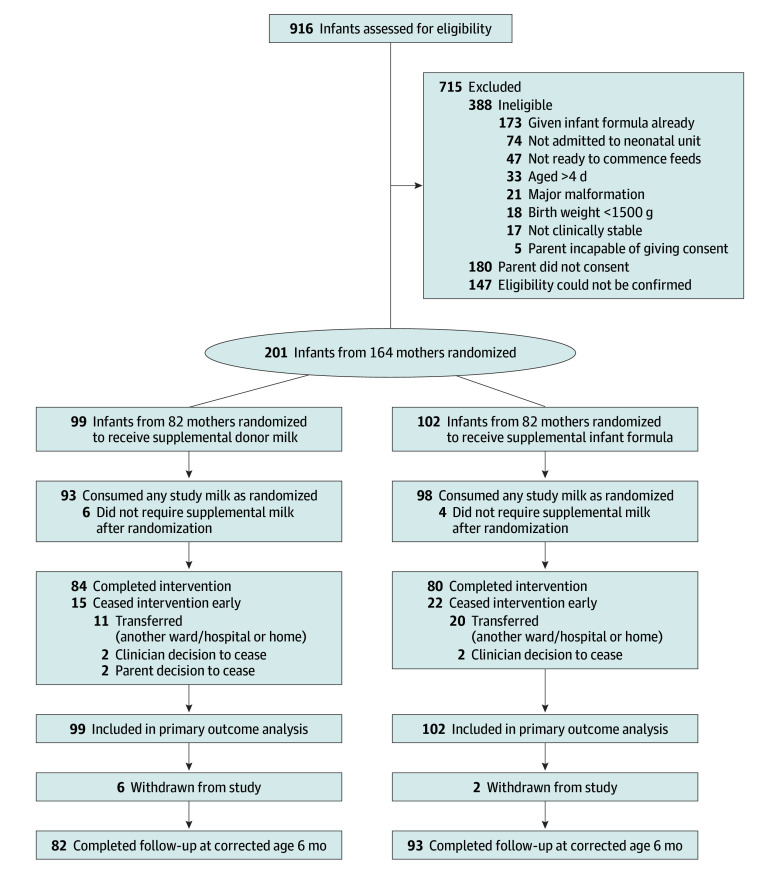
Enrollment, Randomization, and Participant Flow Diagram

**Table 1.** Baseline Characteristics of the Study Population by Treatment Group

Characteristic	No./total No. (%)	
	Donor milk	Formula
**Infant characteristics**		
Infants, No.	99	102
Gestational at birth, mean (SD), wk	34.7 (1.2)	34.6 (1.3)
Infant sex		
Female	44/99 (44.4)	44/102 (43.1)
Male	55/99 (55.6)	58/102 (56.9)
Multiple birth		
Twin	35/99 (35.4)	37/102 (36.3)
Triplet	0/99	3/102 (2.9)
Family history of food allergy	20/95 (21.1)	15/101 (14.9)
Cesarean birth (emergency or planned)	59/99 (59.6)	68/102 (66.7)
Birth weight, mean (SD), g	2258.0 (370.7)	2276.0 (518.5)
Weight *z* score at birth, mean (SD)	−0.2 (0.8)	−0.2 (1.1)
Apgar score at 5 min <7	3/99 (3.0)	5/102 (4.9)
Age at first enteral feed, mean (SD), d	0.3 (0.5)	0.5 (0.7)
Type of milk at first enteral feed		
Maternal EBM	90/98 (91.8)	96/102 (94.1)
Study milk (donor milk or formula)	8/98 (8.2)	6/102 (5.9)
IV dextrose used prior to randomization	88/99 (88.9)	90/102 (88.2)
Age at randomization, mean (SD), d	1.4 (0.8)	1.4 (0.7)
**Maternal characteristics**		
Mothers, No.	82	82
Enrollment site stratum		
WCH	69/82 (84.1)	70/82 (85.4)
RBWH	13/82 (15.9)	12/82 (14.6)
Gestational age stratum, wk		
32 + 0 to 33 + 6	20/82 (24.4)	21/82 (25.6)
34 + 0 to 36 + 6	62/82 (75.6)	61/82 (74.4)
Age at time of birth, mean (SD), y	31.7 (4.6)	31.7 (5.4)
First birth	53/82 (64.6)	47/82 (57.3)
Early pregnancy BMI, mean (SD)^a^	25.8 (5.0)	26.5 (9.1)
Country of birth		
Australia	59/80 (73.8)	59/81 (72.8)
Southern and Central Asia	9/80 (11.2)	5/81 (6.2)
Southeast Asia	3/80 (3.8)	4/81 (4.9)
Other	9/80 (11.3)	13/81 (16.0)
Aboriginal ethnicity^b^	4/82 (4.9)	5/82 (6.1)
Highest level of education		
<High school	3/78 (3.8)	6/81 (7.4)
High school	8/78 (10.3)	6/81 (7.4)
Certificate or diploma	23/78 (29.5)	29/81 (35.8)
University degree	44/78 (56.4)	40/81 (49.3)
Living in an area with the highest index of social disadvantage	12/82 (14.6)	14/82 (17.1)
History of breast milk feeding	21/81 (25.9)	34/81 (42.0)
Preexisting diabetes	2/81 (2.5)	8/80 (10.0)
Gestational diabetes this pregnancy	28/74 (37.8)	14/76 (18.4)
Received antenatal steroids in pregnancy	53/81 (65.4)	54/82 (65.9)
Currently smoking cigarettes or vaping	7/80 (8.8)	6/81 (7.4)
Consumed any alcohol since birth	12/78 (15.4)	10/81 (12.3)
History of asthma	12/80 (15.0)	14/80 (17.5)

Abbreviations: BMI, body mass index; EBM, expressed breast milk; IV, intravenous; RBWH, Royal Brisbane and Women’s Hospital; WCH, Women’s and Children’s Hospital.

^a^ Calculated as weight in kilograms divided by height in meters squared.

^b^ Only Aboriginal ethnicity was collected (Yes/No) and self-reported via questionnaire.

### Study Diet

Infants were randomized at a mean (SD) age of 1.4 (0.8) days, and 191 (95.0%) consumed any study milk (93 of 99 [93.9%] in the donor milk group and 98 of 102 [96.1%] in the formula group; Figure). All participants received the correct study milk allocation. The median (IQR) number of days where study milk was consumed was 6.0 (3.0-8.0) in the donor milk group and 6.0 (3.2-8.0) in the formula group (Table 2). Of 201 infants, 84 (84.8%) and 80 (78.4%) in the donor milk and formula groups, respectively, completed the study intervention period. The reasons for ceasing milk early are outlined in the Figure.

**Table 2.** Study Milk Intake, Feeding Method, and Fortification During Intervention Period

	No./total No. (%)	
	Donor milk (n = 99)	Formula (n = 102)
No. of days where study milk was consumed, median (IQR)	6.0 (3.0-8.0)	6.0 (3.2-8.0)
Total volume of study milk consumed, median (IQR), mL^a^	698.0 (115.2-1136.4)	622.8 (154.8-1148.4)
Total study milk consumed as percentage of total nutrition, median (IQR), %	52.5 (29.6-67.3)	56.4 (36.9-73.5)
Methods of feeding study milk		
Gavage	88/99 (88.9)	93/102 (91.2)
Bottle	19/99 (19.2)	31/102 (30.4)
Perfusor	18/99 (18.2)	22/102 (21.6)
Syringe	2/99 (2.0)	5/102 (4.9)
Other method	1/99 (1.0)	0/102
Maternal milk fortified	27/99 (27.3)	29/102 (28.4)
Extra calories ordered^b^	18/99 (18.2)	23/102 (22.5)
Other supplements used^c^	23/99 (23.2)	28/102 (27.5)
Required supplementary milk feeds after intervention period	41/98 (41.8)	46/102 (45.1)

^a^ The volume of study milk consumed during study day was unable to be recorded on 14 occasions, affecting 13 infants.

^b^ Intervention group received fortified donor milk and control group received preterm formula.

^c^ Iron, milk thickener, multivitamins, etc.

The median (IQR) total study milk consumed as a percentage of total milk consumed during the intervention period was 52.5% (29.6%-67.3%) in the donor milk group and 56.4% (36.9%-73.5%) in the formula group.

During the intervention period, maternal milk was fortified for 27 infants in the donor milk group (27.3%) and 29 in the formula group (28.4%). Extra calories were ordered for 18 infants in the donor milk group (18.2%, resulting in receipt of fortified donor milk) and 23 in the formula group (22.5%, resulting in receipt of preterm formula). Blinding was successful based on parental responses (eTable 1 in Supplement 3).

### Outcomes

The mean (SD) time to reach full enteral feeds in days did not differ between groups (donor milk group: 5.7 [2.6] days; formula group: 5.8 [3.4] days; adjusted mean difference, −0.07; 95% CI, −0.90 to 0.76) (Table 3). Conclusions did not change in post hoc sensitivity analyses (eTables 2-4 in Supplement 3).

**Table 3.** Time to Reach Full Enteral Feeds (Primary Outcome), In-Hospital Weight Outcomes, and Length of Stay

Outcome	Mean (SD)		Treatment effect (95% CI)^a^
	Donor milk (n = 99)^b^	Formula (n = 102)^b^	
Primary outcome: time to reach full enteral feeds, d	5.7 (2.6)	5.8 (3.4)	−0.07 (−0.90 to 0.76)^c^
Secondary outcomes			
Time to reach full suck feeds, d^d^	18.5 (11.0)	18.4 (10.4)	0.86 (0.58 to 1.30)
No. episodes feeding intolerance (during intervention)^e^	0.6 (3.1)	0.5 (1.9)	1.44 (0.38 to 5.37)
No. episodes feeding intolerance (randomization to discharge)^e^	1.6 (8.8)	0.7 (3.0)	1.66 (0.38 to 7.32)
Duration of use of IV glucose (randomization to discharge), d	2.0 (1.2)	2.1 (1.6)	−0.08 (−0.5 to 0.34)
Duration of use of parenteral nutrition (randomization to discharge), d	0.2 (1.5)	0.0 (0.5)	0.20 (−0.11 to 0.51)
Time until birth weight regained, d^d^	10.7 (5.7)	8.4 (4.4)	0.65 (0.47 to 0.88)
Weight *z* score at discharge	−1.0 (0.8)	−0.8 (1.0)	−0.15 (−0.43 to 0.12)
Change in weight *z* score (discharge − birth)	−0.7 (0.6)	−0.6 (0.5)	−0.09 (−0.25 to 0.08)
Length of hospital stay (including time in early discharge programs), d	21.4 (11.5)	21.2 (11.4)	0.66 (−2.48 to 3.81)
Length of hospital stay (excluding early discharge programs), d	17.7 (10.2)	18.2 (11.0)	−0.20 (−3.11 to 2.71)

Abbreviation: IV, intravenous.

^a^ Treatment effect is a difference in means (donor milk − formula) adjusted for site, gestational age, and multiple birth unless otherwise indicated.

^b^ Based on complete cases (no missing data for primary outcome of days until full enteral feeds, maximum 1 missing infant in each group for secondary outcomes).

^c^ *P* = .87.

^d^ Treatment effect is a hazard ratio (donor milk vs formula) adjusted for site, gestational age, and multiple birth.

^e^ Treatment effect is an incidence rate ratio (donor milk vs formula) adjusted for site, gestational age, and multiple birth.

There was no difference between groups in the incidence of feeding intolerance (during or after the intervention period), duration of use of IV glucose or parenteral nutrition, or the time to reach full suck feeds. Infants in the donor milk group had a lower rate of birth weight regain than those in the formula group (hazard ratio, 0.65; 95% CI, 0.47-0.88), which equated to a 2-day longer period to regain birth weight on average. There was no difference between groups in weight *z* score at discharge or change in weight *z* score (from birth to discharge). There were no cases of sepsis or NEC. Length of hospital stay was similar between groups (Table 3).

The 6-month CA questionnaire was completed for 175 infants (87.1%); however, not all families attended the in-person visits postdischarge. Complete infant weight measurements were available for 100%, 77.6%, and 75.6% of participants at discharge, 2-, and 4-month CA, respectively. There were no differences between groups in mean infant weight, length, head circumference, and percentage fat-free mass from discharge to 4-month CA (Table 4). At discharge, 72 infants (72.7%) and 65 infants (63.7%) were exclusively fed breast milk (including direct breastfeeding and expressed breast milk) in the donor milk and formula groups, respectively.

**Table 4.** Secondary Outcomes: Growth, Body Composition, Breastfeeding, and Hospital Readmissions to 6-Month Corrected Age (CA)

Outcome	Mean (SD)		Treatment effect (95% CI)^a^
	Donor milk (n = 99)^b^	Formula (n = 102)^b^	
**Infant weight, kg**			
Discharge	2.5 (0.3)	2.6 (0.5)	−0.13 (−0.25 to 0.00)
2-mo CA	5.4 (0.7)	5.3 (0.9)	0.01 (−0.25 to 0.28)
4-mo CA	6.6 (0.7)	6.6 (1.0)	−0.04 (−0.34 to 0.26)
**Infant length, cm**			
Discharge	46.2 (2.1)	46.8 (2.6)	−0.47 (−1.30 to 0.36)
2-mo CA	58.0 (2.7)	58.0 (3.3)	−0.20 (−1.28 to 0.87)
4-mo CA	62.8 (3.0)	62.9 (2.9)	−0.20 (−1.29 to 0.89)
**Infant head circumference, cm**			
Discharge	33.0 (1.2)	33.2 (1.6)	−0.12 (−0.62 to 0.37)
2-mo CA	39.6 (1.5)	39.6 (1.7)	−0.05 (−0.56 to 0.47)
4-mo CA	41.9 (1.4)	42.0 (1.5)	−0.07 (−0.55 to 0.42)
**Infant fat-free mass, %**			
Discharge	84.5 (8.0)	84.9 (6.3)	−0.33 (−3.22 to 2.55)
2-mo CA	75.5 (3.9)	74.6 (4.8)	0.64 (−1.43 to 2.70)
4-mo CA	74.3 (4.4)	75.0 (4.2)	−0.71 (−2.60 to 1.18)
**Exclusive breast milk feeding^c^**			
Discharge, No./total No. (%)	72/99 (72.7)	65/102 (63.7)	1.05 (0.89 to 1.24)
2-mo CA, No./total No. (%)	34/77 (44.2)	26/85 (30.6)	1.33 (0.89 to 1.98)
4-mo CA, No./total No. (%)	25/83 (30.1)	21/90 (23.3)	1.25 (0.76 to 2.04)
6-mo CA, No./total No. (%)	2/82 (2.4)	1/93 (1.1)	Not estimated
**Any breast milk feeding^c^**			
Discharge, No./total No. (%)	94/99 (94.9)	96/102 (94.1)	1.00 (0.94 to 1.08)
2-mo CA, No./total No. (%)	56/77 (72.7)	63/85 (74.1)	0.98 (0.81 to 1.18)
4-mo CA, No./total No. (%)	52/83 (62.7)	50/90 (55.6)	1.13 (0.88 to 1.45)
6-mo CA, No./total No. (%)	43/82 (52.4)	48/93 (51.6)	1.02 (0.76 to 1.35)
No. of hospital readmissions (discharge to 6-mo CA)^d^	0.3 (0.6)	0.2 (0.6)	1.42 (0.63 to 3.20)
Total length of hospital readmissions, d	0.4 (0.9)	0.4 (1.2)	0.06 (−0.34 to 0.45)

^a^ Treatment effect is a difference in means (donor milk − formula) adjusted for site, gestational age, and multiple birth unless otherwise indicated.

^b^ Based on complete cases (between 0 and 98 missing values total at discharge, 39 and 112 at 2-month CA, and 28 and 105 at 4-month CA).

^c^ Treatment effect is an unadjusted relative risk (donor milk vs infant formula) due to convergence problems with the adjusted model.

^d^ Treatment effect is a ratio of means (donor milk vs infant formula) adjusted for site, gestational age, and multiple birth.

## Discussion

Among moderate to late preterm infants with insufficient maternal milk available, the use of supplementary donor milk compared with formula did not alter the time to reach full enteral feeds, which, for most infants, was achieved within 7 days of enrollment. In addition, the frequency of feed intolerance, use of IV glucose, and time to reach full suck feeds were similar between groups. Our findings suggest that the type of milk supplement does not affect feeding progression in this preterm population and are consistent with a recent trial, which found no difference in time to full enteral feeds between those given a milk supplement (predominantly formula) compared with those fed exclusively maternal milk.^29^

In very preterm infants, donor milk has been associated with slower weight gain in hospital.^16,30^ In our trial, infants fed donor milk took on average 2 days longer to regain their birth weight. These effects appear to be transient; by discharge, the groups were similar in weight *z* score, change in weight *z* score, and weight at 2- and 4-month CA. Further, length and head circumference were similar between groups at all time points. We identified only 1 other randomized clinical trial examining supplemental pasteurized donor milk as an alternative to formula in more mature preterm infants.^31^ Similar to our findings, Pithia and colleagues^31^ reported weight and length were comparable between groups at all time points among the participating 32 infants. The exception was head circumference *z* score, which was greater in formula-supplemented infants at 6 to 8 weeks’ postnatal age.

Rates of exclusive breast milk feeding were somewhat higher in the donor milk group at discharge and up to 4-month CA, whereas rates of any breast milk feeding were similar between groups. Pithia and colleagues^31^ report similar rates of exclusive breastfeeding at 6 to 8 weeks’ postnatal age in donor milk and formula-supplemented infants. Observational studies in moderate to late preterm infants report conflicting findings on the impact of supplemental donor milk on exclusive breastfeeding rates, ranging from potential benefit to no clear difference.^22^ However, most have methodological limitations, including small sample sizes. Further research examining the effect of donor milk as a breastfeeding support strategy is needed, particularly as there is growing use of donor milk in well newborn nurseries.^17^

Previous research has demonstrated that early life growth and body composition in preterm infants influence both later cognitive development and metabolic health.^32,33^ Our trial found treatment groups were similar in body composition, including percentage fat-free mass, either at discharge or at 2- or 4-month CA. A previous observational study of feeding practices in moderate to late preterm infants found exclusive breastfeeding was associated with lower fat mass and lean body mass at term-equivalent age and greater lean mass gain at 3-month CA compared with mixed or exclusive formula feeding.^34^ In contrast, Alexander and colleagues^29^ reported a similar percentage of fat mass at 4-month CA among infants receiving a milk supplement compared with maternal milk only. However, their findings are not directly comparable to our trial, as the milk supplement group included infants fed either donor milk or formula.

Our finding of no clear effect of donor milk on breastfeeding or body composition may be explained by the high use of supplemental formula after the intervention period ceased (41.8% and 45.1% in the donor milk and formula groups, respectively). We designed the trial to provide short-term supplemental milk to support a transition to maternal milk feeds as quickly as possible and to avoid supplemental milk inadvertently discouraging breastfeeding. However, it is possible that the 8-day period provided insufficient time to establish a full milk supply, as preterm birth can disrupt milk production.^12^ Further trials of donor milk with an extended intervention period are warranted in moderate to late preterm infants. Future trials should consider longer-term impacts, including neurodevelopment, an important treatment goal of neonatal nutritional interventions. Further, an assessment of economic impacts is warranted, as donor milk is significantly more expensive than formula but may have economic benefits, particularly if found to improve exclusive breastfeeding in this large preterm population. A large trial is now underway (ACTRN12624000289516).

### Strengths and Limitations

This trial has several strengths. It was a multisite study and the largest randomized clinical trial to date assessing supplemental donor milk in moderate to late preterm infants. Clinicians, families, and outcome assessors were successfully blinded to group allocation, reducing the chance of bias arising in the clinical management or data collection. Protocol deviations, including unblinding, were rare (eAppendix 2 in Supplement 3). Primary outcome data were available for all infants.

Nevertheless, the trial has limitations. Not all families participated in the in-person assessment at discharge, 2-, and 4-month CA, reflecting hospital visiting restrictions and family preferences during the coronavirus pandemic. Our trial only included infants born between 32 + 0 and 36 + 6 weeks who were admitted to a neonatal unit. Therefore, findings may not be generalizable to moderate to late preterm infants who do not need specialized care. Finally, the control group was given a term formula, as this was standard practice in both sites. However, preterm formula is also used in this population.^10^

## Conclusions

In this randomized clinical trial, among moderate to late preterm infants with insufficient maternal milk available, supplemental donor milk did not reduce time to full enteral feeds compared with term infant formula for a period of up to 8 days or demonstrate other clear benefits to 6-month CA. Larger trials with longer follow-up are needed.

## Notes

Supplement 1. Trial Protocol

Supplement 2. Statistical Analysis Plan

Supplement 3. eTable 1. Which Group Mothers Thought Their Baby Was In, by Treatment Group eAppendix 1. Sensitivity Analyses for the Primary Outcome eTable 2. Time to Full Enteral Feeds, by Treatment Group, With Additional Adjustment for Gestational Diabetes eTable 3. Time to Full Enteral Feed, by Treatment Group, Restricted to Eligible Infants eTable 4. Time to Full Enteral Feed, by Treatment Group, Restricted to Infants Born ≥34 Weeks’ Gestational Age eAppendix 2. Protocol Deviations eReference.

Supplement 4. Data Sharing Statement
