# Supplementary material for: Supplemental Donor Milk vs Infant Formula in Moderate to Late Preterm Infants: A Randomized Clinical Trial
Source: JAMA Pediatr. 2025 Aug 4;179(10):1065–73. doi: 10.1001/jamapediatrics.2025.2365 (PMC12322819; doi:10.1001/jamapediatrics.2025.2365)
Supplement: Supplement 1. — Trial Protocol [file jamapediatr-e252365-s001.pdf]

PROTOCOL

Effect of supplementary pasteurised donor human milk compared with standard formula on time to full enteral feeds, feed tolerance, growth, breastfeeding and length of hospital stay in moderate and late preterm babies – a pilot randomised controlled trial

LAY TITLE: Giving donor milk instead of formula in moderate-late preterm infants: the GIFT trial

Protocol version: Version 1.3, 25th November 2021

Sponsoring Institution: South Australian Health and Medical Research Institute (SAHMRI)

STATEMENT OF COMPLIANCE

This document is a protocol for a randomised controlled trial. The study will be conducted in compliance with all stipulations of this protocol, the conditions of ethics committee approval, the NHMRC National Statement on Ethical Conduct in Human Research (2007 – Updated March 2014) and the Integrated Addendum to ICH E6(R1): Guideline for Good Clinical Practice ICH E6(R2).

I agree that the study will be conducted in accordance with the conditions outlined in the protocol (subject to any amendments).

I understand that the information in this protocol is confidential. Publication of information related to this protocol in formats including, but not limited, conference presentations, journal articles, public reports and internet postings, must be submitted to the trial steering committee for consideration within a reasonable time frame of any due dates. Approval for all said activities must have the written permission of the chair of the steering committee or delegate prior to the event.

Investigator's Name: Alice Rumbold

Investigator's Signature:

Date: 30/03/2022

Study site: WCH & RBWH

Chair of the Steering Committee: Associate Professor Alice Rumbold

Signature:

Date: 30/03/2022

1. Contents

2. PROTOCOL SUMMARY **4**

3. LIST OF ABBREVIATIONS **5**

4. INVESTIGATORS **6**

5. BACKGROUND AND RATIONALE **1**

6. STUDY OBJECTIVES **3**

6.1 Primary objective **3**

6.2 Secondary objectives **3**

7. METHODS **5**

7.1 Study setting **5**

7.1 Study design **5**

7.3 Expected duration **5**

7.4 Eligibility criteria **5**

7.5 Study treatments **6**

7.5.1 Intervention group (intervention nutrition) 6

7.5.2 Control group (control nutrition) 7

7.6 Preparation of intervention and control nutrition **7**

7.7 Administration of study nutrition **8**

7.8 Discontinuation of study nutrition **9**

7.9 Compliance **10**

7.10 Outcome measures **10**

7.11 Data collection **10**

7.11.1 At enrolment 10

7.11.2 During intervention period 11

7.11.3 At discharge and study follow-up assessments 11

7.11.4 Use of linked administrative data 12

7.11.5 Assessment of lactation support and overall breastfeeding rates 13

7.12 Data management **13**

7.13 Sample size **13**

7.14 Statistical methods **14**

7.15 Data and safety monitoring **14**

7.16 Ethics, recruitment and dissemination **15**

7.16.1 Modifications of the protocol 15

7.16.2 Protocol deviations 15

7.16.3 Consent 16

7.16.4 Confidentiality 16

7.16.5 Use of data and publication policy 17

8. REFERENCES **18**

9. APPENDIX 1 .... **19**

2. PROTOCOL SUMMARY

**Title**	Effect of supplementary pasteurised donor human milk compared with standard formula on feed tolerance, growth, breastfeeding and length of stay in moderate and late preterm babies – a pilot randomised controlled trial.
**Objectives**	To compare the effect of supplementary pasteurised donor human milk versus standard infant formula on time to establish full enteral feeds, feed intolerance, growth, breastfeeding and length of stay in clinically well preterm infants born between 32+0 and 36+6, with a birth weight ≥1500g and with insufficient maternal breast milk available.
**Design**	Blinded, randomised controlled trial.
**Population**	Infants born between 32+0 and 36+6 completed weeks' gestation; birth weight ≥ 1500g; admitted to the neonatal unit; clinically stable; ready to commence enteral feeds or commenced enteral feeds but with insufficient maternal breast milk and ≤ 4 days old.
**Outcomes**	Primary: Time to full enteral feeds (days), defined as 150ml/kg/d.<br><br>Secondary: Number of episodes of feed intolerance, defined as the decision to stop or reduce oral feeds, growth parameters at discharge, 2, and 4 months' corrected age (CA) (weight z-score for gestation age and z-score change, weight, length and head circumference), body composition at discharge, 2 and 4 months' CA, breast milk feeding (fully or any) at discharge, 2, 4 and 6 months' CA, length of hospital stay, and neonatal complications (e.g. late onset sepsis).
**Intervention**	Pasteurised donor human milk provided by Australian Red Cross Lifeblood. All milk will undergo microbiological screening before and after Holder pasteurisation (30 minutes at 62.5°C) according to Lifeblood policies.
**Control**	Standard term formula.
**Duration**	The supplementary nutrition will be commenced as per unit protocols per gestational age (ml/kg/day) up to 160-180 ml/kg/day. The intervention period will cease on study day 8 (day supplementary feeds started + 7 days), or earlier if maternal expressed breast milk supply is sufficient to feed at a rate of 150ml/kg/day or if discharged home. Infants will be followed up for growth and feeding outcomes until 6 months' corrected age.
**Sample size**	200 infants.

3. LIST OF ABBREVIATIONS

CA	Corrected age
EBM	Expressed breast milk
HREC	Human Research Ethics Committee
Lifeblood	Australian Red Cross Lifeblood
NEC	Necrotising enterocolitis
NICU	Neonatal intensive care unit
PDHM	Pasteurised donor human milk
SAE	Serious adverse events

4. INVESTIGATORS

COORDINATING CENTRE

A/Prof Alice Rumbold (Principal Investigator)
Principal Research Fellow
South Australian Health and
Medical Research Institute (SAHMRI)
72 King William Road
North Adelaide SA 5006
Phone: 08 8128 41940
Mobile: 0411269831
Email: alice.rumbold@sahmri.com

Dr Lisa Yelland
NHMRC Postdoctoral Research Fellow
and Senior Statistician, SAHMRI
72 King William Road
North Adelaide SA 5006
Ph: 08 8128 4443
Email: lisa.yelland@adelaide.edu.au

A/Prof Carmel Collins
Principal Research Fellow
SAHMRI
72 King William Road,
North Adelaide, SA 5006
Phone: 8128 4409
Email: carmel.collins@sahmri.com

Prof Maria Makrides
Theme Leader
SAHMRI
72 King William Road,
North Adelaide, SA 5006
Phone: 8128 4416
Email: maria.makrides@sahmri.com

STUDY SITES

Dr Andy McPhee
Staff Neonatologist, Neonatal Medicine
Women's and Children's Hospital
72 King William Rd,
North Adelaide SA 5006
Phone: 8161 7631
Email: andrew.mcphee@sa.gov.au

A/Prof Amy Keir
Staff Neonatologist, Neonatal Medicine
Women's and Children's Hospital and SAHMRI
72 King William Road,
North Adelaide, SA 5006
Phone: 08 8161 7631
Email: amy.keir@adelaide.edu.au

Dr Pieter Koorts
Director of Neonatology, Women's and Newborn Services
Level 5, NHB, Royal Brisbane & Women's Hospital
Butterfield St, Herston, Qld 4029
Phone: 07 3646 8918
Email: Pieter.koorts@health.qld.gov.au

Dr Melissa Lai
Staff Neonatologist, Women's and Newborn Services
Level 5, NHB, Royal Brisbane & Women's Hospital
Butterfield St, Herston, Qld 4029
Phone: 07 3646 8918
Email: Melissa.lai@health.qld.gov.au

Ms Karen Langford
Clinical Nurse Consultant, Queensland Milk Bank
Level 6, NHB, Royal Brisbane & Women's Hospital
Butterfield St, Herston, Qld 4029
Phone: 07 3646 0542
Email: karen.langford@health.qld.gov.au

Ms Lyn Chapple
Clinical Nurse, Grantley Stable Neonatal Unit
Level 5, NHB, Royal Brisbane & Women's Hospital
Butterfield St, Herston, Qld 4029
Phone: 07 3646 8918
Email: Lynette.chapple@health.qld.gov.au

A/Prof Tim Donovan
Senior Staff Neonatologist, Women's and Newborn Services
Level 5, NHB, Royal Brisbane & Women's Hospital
Butterfield St, Herston, Qld 4029
Phone: 07 3646 8918
Email: Tim.Donovan@health.qld.gov.au

AUSTRALIAN RED CROSS LIFEBLOOD

Dr Laura Klein
Lifeblood Milk Research Fellow
Business Growth and Innovation
Australian Red Cross Lifeblood
17 O'Riordan St
Alexandria NSW 2015
Phone: +61 2 9234 2361
Email: LKlein@redcrossblood.org.au

5. BACKGROUND AND RATIONALE

Inadequate nutrition at critical stages of early life can have lasting effects on the developing brain and organ systems. This is well characterised in the very preterm infant (born <32 weeks' gestation), with evidence that suboptimal energy and protein intakes, even in the first week of life, increase the risk of neonatal complications and adverse neurodevelopmental outcomes[1]. This has led to a significant focus on intense early nutritional support of very preterm infants in the neonatal nursery.

In contrast, there has been limited examination of optimal nutritional practices in infants born moderate to late preterm, between 32 and 36 weeks' gestation. Each year in Australia over 21,000 infants are born moderate to late preterm[2]. These infants account for the majority of preterm births (>80%) and a significant proportion of neonatal nursery workload.

Moderate and late preterm infants have poorer health and developmental outcomes than infants born at term. Risks of respiratory distress, hypoglycaemia, jaundice, infections and faltering growth[3,4] are increased, contributing to a prolonged neonatal hospital stay and increased risk of readmission in the first 6 months of life. In the longer term, moderate and late preterm infants have an increased risk of cognitive, motor and behavioural difficulties, and poorer metabolic health (e.g. obesity and hypertension)[5]. These adverse outcomes have profound impacts on children and their families, with significant financial impact on the health and educational systems[6].

Poor early nutrition is hypothesised to contribute to many of the short- and long-term complications seen in moderate and late preterm infants. These infants frequently experience delays establishing full enteral feeds due to poor coordination of sucking and breathing mechanisms and immature gut motility. In addition, mothers of preterm infants often struggle to produce enough breast milk, due to illness and separation from the baby. As a result, over 80% of moderate and late preterm infants require supplementary nutrition, especially in the critical first few weeks after birth[5].

There is little high-quality research evaluating initiatives to improve nutrition in moderate to late preterm infants and no dietary guidelines specific for this group[5,7]. Management practices in neonatal units across Australia and New Zealand are highly variable[8]. Common practices include provision of supplementary infant formula and/or intravenous fluids until mother's own milk is available and full milk feeds are tolerated. Unpublished pilot data from our own research with late preterm infants (n=270) born at the Women's and Children's Hospital between 2017 and 2019, indicates use of supplementary infant formula during the neonatal admission is very high. For example, use of term formula during the admission was

documented in 62% of late preterm infants, and 22% had formula as their first milk feed. Only 36% of infants were exclusively breastfeeding at 6 weeks corrected age.

Use of infant formula in this population brings a number of challenges. Due to the immaturity of their gut, preterm infants often have difficulties tolerating and digesting the foreign proteins in formula. Consequently, use of formula can contribute to feed intolerance (repeated vomiting causing oral feeds to be stopped or reduced), increasing the risk of dehydration and need for intravenous fluids, in turn creating a risk of infection and prolonged hospital admission. Formula also does not contain the many protective bioactive compounds that are found only in human milk that promote immune system development and aid digestion of nutrients[9]. These compounds are especially critical for preterm infants. In addition, early use of formula, even as a supplement to mother's own milk, increases the risk of early cessation of breastfeeding, as it can reduce demand for breastmilk and undermine confidence in breastfeeding[10,11].

The most promising solution to improve nutrition in this group is to provide pasteurised breast milk from another mother, known as pasteurised donor human milk (donor milk). Currently, donor milk is routinely given to supplement feeds in very preterm infants <32 weeks and those with a birth weight <1500 g, based on high quality evidence of a reduction in the risk of necrotising enterocolitis (NEC)[12]. Trials also show that, compared with formula, donor milk improves feed tolerance in very preterm infants but is associated with slower rates of weight gain and linear growth in hospital. Whether this reflects impaired growth is unclear, as formula may promote growth in fat mass at the expense of lean muscle mass, suggesting poorer overall quality of growth[13].

Donor milk may also be an important strategy to support continued maternal breastmilk feeds and breastfeeding. This may occur through early avoidance of formula and its potential detrimental effects on the immature gut epithelium, enabling a faster transition to full milk feeds. In addition, when used as part of ongoing lactation support, donor milk may contribute to supporting a culture of exclusive human milk feeding in the neonatal unit. Evidence suggests that donor milk is associated with higher rates of any breastfeeding on discharge[14]. However, the effects on rates of exclusive breastfeeding at discharge are less consistent, indicating the need to continue to promote and monitor lactation support in units that have access to donor milk.

There are no high-quality studies examining use of donor milk in moderate and late preterm infants. In a systematic review of the literature we identified three trials, all with major methodological limitations. All were undertaken in the 1970s, thus their relevance to contemporary neonatal care is very limited. The trials involved 131 moderate to late preterm

infants in total, with 44 allocated to donor human milk. The only outcomes reported were growth-related and findings were inconsistent, with trials reporting no differences in weight gain or linear growth[15] slower weight gain in donor milk fed infants[16] and higher weight gain with donor milk[17].

While donor milk is likely to confer benefits to moderate and late preterm infants, the benefits need to be carefully examined and weighed against the costs. Donor milk costs more to provide than infant formula, so expanding availability to all preterm infants could substantially increase healthcare costs for infants if it does not have the expected health benefits.

Historically, in Australia donor milk has been prioritised for babies born <32 weeks' gestation due to limited supplies. With the recent establishment of the Australia Red Cross Lifeblood milk service, there is now a stable, safe supply of donor milk in Australia. This provides a unique opportunity to examine whether donor milk can reduce nutrition-related complications in moderate and late preterm infants. We will undertake a pilot randomised trial to examine this focussing on the time to establish full enteral feeds, feed intolerance and other important clinical outcomes up to 6 months' corrected age (CA). This will provide crucial initial evidence to inform the design of a larger trial powered to examine the impact of donor milk on neonatal length of stay and serious neonatal complications.

6. STUDY OBJECTIVES

6.1 Primary objective

To compare the effect of pasteurised donor human milk versus standard infant formula, used to supplement insufficient maternal breast milk, on the time to establish full enteral feeds in clinically well preterm infants born between 32+0 and 36+6 weeks' gestation, with a birth weight ≥1500g.

6.2 Secondary objectives

To compare the effect of pasteurised donor human milk versus standard infant formula in this study population, on:

- Feeding intolerance, defined as the decision to delay or stop enteral feeds
- Time to establish full suck feeds
- Weight, length and head circumference gain in hospital and up to 4 months' CA
- Neonatal complications including proven late onset sepsis, proven necrotising enterocolitis
- Body composition at discharge, 2 and 4 months' CA

- Frequency and duration of breast milk feeds (fully or any)
- Length of initial hospital stay, frequency and length of any readmissions

To generate preliminary evidence of effects of pasteurised donor human milk on clinical outcomes in preterm infants born between 32+0 and 36+6 weeks' gestation, to inform the design of a larger randomised trial, powered to detect changes in length of hospital stay and readmissions.

7. METHODS

7.1 Study setting

Infants will be recruited from the neonatal units of the Women's and Children's Hospital, South Australia and Royal Brisbane and Women's Hospital, Queensland.

7.1 Study design

This is a multi-centre, blinded randomised controlled trial with two parallel groups, followed up until 6 months' CA. Infants will be randomly allocated to either the intervention or control group with a 1:1 allocation using a secure web-based randomisation service (on REDCap). Stratification will occur for centre and gestational age (≤33 weeks, ≥34 weeks). A computer-generated randomisation schedule using balanced variable block design will be prepared and held by an independent statistician, not otherwise involved in the trial. Twins will be co-randomised to the same treatment arm. The randomisation system will be housed on SAHMRI secure servers.

Participants, families, clinical teams and research staff, including research nurses, outcome assessors and data analysts will be blinded to the randomisation groups. The processes for maintaining blinding are described in 5.6.

Parents will be approached about the study by the research nurse where possible antenatally, or as soon as possible after birth when the infant is clinically stable. Written informed consent will be obtained from those interested in participating. Randomisation will occur at the time when the clinical decision is made to supplement maternal milk, that is, when mother's milk is deemed insufficient to meet the infant's enteral requirements.

7.3 Expected duration

Recruitment will commence in July 2021. A recruitment rate of at least 2-3 infants per week per site is anticipated, therefore, recruitment is expected to be completed by the end of 2021.

7.4 Eligibility criteria

Eligible infants must meet all of the following criteria:

- 32+0 and 36+6 completed weeks' gestation at birth;*

- Birth weight ≥ 1500g;
- Admitted to the neonatal unit**;
- Clinically stable;
- Ready to commence enteral feeds or commenced enteral feeds with human milk but insufficient maternal breast milk is available;
- Aged ≤ 4 days old;
- Has a parent or guardian capable of giving informed consent.

*Only infants with a gestation of ≥34 weeks at birth will be enrolled at the Royal Brisbane and Women's Hospital.

**If it is anticipated that an infant will be transferred to the postnatal ward quickly then they will not be eligible for the study, as for practical reasons the study nutrition will not be able to administered on the postnatal ward.

An infant who meets any of the following criteria will be excluded from participation:

- Metabolic disorder that precludes breastfeeding;
- Major congenital malformation either likely to interfere with the ability to ingest milk or requiring surgery in the first 6 months of life (e.g. complex congenital heart disease);
- Given infant formula prior to randomisation.

Infants of mothers' who do not intend to breastfeed for either medical or social reasons will be eligible but excluded from analyses of method of feeding (at discharge and up to 6 months' CA).

7.5 Study treatments

Eligible infants will be randomised to either the intervention or control group. The study treatments will be administered to infants in the neonatal unit.

7.5.1 Intervention group (intervention nutrition)

The intervention consists of pasteurised donor human milk provided by Australian Red Cross Lifeblood Milk. All milk will undergo microbiological screening and Holder pasteurisation (30 minutes at 62.5°C) according to Australian Red Cross policies. All milk donors are screened in line with international milk banking guidelines.

7.5.2 Control group (control nutrition)

The control consists of standard term formula, unless extra calories are ordered by the attending physician or neonatal nurse practitioner, in which case infants in the control group will be changed to preterm formula.

7.6 Preparation of intervention and control nutrition

Aliquots of mother's own milk be ordered by the attending physician or neonatal nurse practitioner and prepared as per local unit protocols. When the medical decision is made to use supplementary nutrition, the infants will be randomised by the research nurse and their treatment group allocation colour will be recorded in the infant's case notes and feeding/fluid balance chart.

The intervention nutrition will be provided by Australian Red Cross Lifeblood in standard bottles and stored as per current practice until ready to be made up as study nutrition.

At the Royal Brisbane Women's Hospital, each day the assistant in nursing staff (AINs) will make up study nutrition in bottles of either 120ml or 60 ml that are covered with tape to maintain blinding, with a colour-coded sticker added and capped with a bottle transfer lid. A stock of opaque syringes will be provided with the bottles for nursing staff to draw up study nutrition for each feed. The transfer lid allows study nutrition to be drawn up without opening the bottle and risking unblinding. As it is standard care at this site that each infant has their own fridge next to their cot, the bottles and syringes will be delivered directly to the fridge at the cotside. Nursing and midwifery staff on the ward will take an opaque syringe, draw up milk from the bottles and administer the study nutrition as needed.

The trial will use standard food grade syringes and bottles, covered completely with an opaque label and a small coloured sticker. To reduce the risk of unblinding, 2 separate colour-coded treatment groups for donor milk will be prepared, and two separate colour-coded control groups will be prepared (hence a total of four colour coded groups). Syringes and bottles will be labelled with the infant's name, Study Nutrition Group <colour>, a batch number, expiry date and time. The study nutrition will be made up in the neonatal unit's milk room with the door closed.

At the Women's and Children's Hospital the study nutrition will be made up by a food services staff member, not involved in the recruitment or follow up of participants. Study nutrition will be ordered via the current process for ordering aliquots of mother's own milk, made by the attending physician or neonatal nurse practitioner. The food services member will be aware of the study group allocation and make up the colour coded aliquot in the required volume then adding a label with the baby's name and UR number. These will be

delivered to the wards and administered by nursing and midwifery staff as per usual practice. An additional stock of study nutrition in each colour coded group will be made up daily and kept in the milk room to be available for babies that may be randomised after hours and after the milk orders have been filled for that day.

If during the study intervention period, a decision is made by the treating health care team to give extra calories, there will be no restrictions on use of human milk fortifier. To maintain blinding, food services staff will be asked to fortify mother’s own milk first. If there is insufficient volume of mother’s own milk to add fortifier, babies in the donor milk group will have fortifier added to the study nutrition. For babies in the control group, the study nutrition will be changed to preterm formula. We anticipate the number of babies who will require fortification during the study period will be small.

At each site, the staff involved in preparing the study nutrition will maintain a list of stock (group allocation, batch number and expiry date) prepared each day.

The batch number listed on donor milk aliquots will be the same batch number listed on the bottles provided by Lifeblood. A list of batch numbers containing the same number of digits will be generated for use on the formula (control group) aliquots. The expiry date will be listed as 24 hours after the aliquot preparation.

Aliquots will be delivered to the wards and stored at 4°C in the fridge in separate containers per group colour.

The batch number allocated and administered to each infant will be recorded either on the feeding chart or as per standard practice at each unit and current Lifeblood requirements for tracking donor milk batches.

Research nurses will maintain daily checks of study nutrition use, including adherence to treatment group allocation, a daily stock take and discard aliquots that have passed their expiry date which is 24 hours after defrosting/being made up.

7.7 Administration of study nutrition

The intervention and control nutrition will be commenced enterally at a volume consistent with unit protocols for gestational age to a maximum of 160-180 ml/kg/day, accounting for any parenteral intake. Feeds will be advanced at 10-30 mls/kg/day as per local protocols and the discretion of the treating team (see nutrition protocol summary in APPENDIX 1).

Infants in both groups will be fed first with the mother's own expressed breast milk (EBM), where available. It is expected that the volume of study nutrition required will decrease over the intervention period as the mother's own milk supply increases.

During the intervention period, use of formula that is not part of the study nutrition will not be permitted. There will be no restrictions on the use of IV fluids (glucose and/or electrolytes, PNS or lipids), medications (e.g. dextrose gel) or additional nutrition (e.g. pentavite) with the exception of infant formula.

The intervention will cease on day 8 following randomisation or earlier if there is sufficient mother's own milk to support the target fluid intake, or the infant is discharged from hospital.

After the intervention period has ceased, if the infant still requires supplementary nutrition they will be fed according to local unit protocols, with no restrictions on use of formula, or fortification of mother's own EBM.

7.8 Discontinuation of study nutrition

The clinical management of participants will be under the direction of the attending neonatologist. If, in the clinical opinion of the attending neonatologist, an infant is not tolerating enteral feeds and requires parenteral nutrition support then the infant will be managed according to local protocols regarding central venous catheter insertion and management. The decision to cease study nutrition will be made by the attending neonatologist.

Parents/guardians may have the study nutrition discontinued or withdraw their child from the study for any reason at any time, with no effect on their clinical care.

All randomised infants who are prematurely discontinued from the study nutrition will be considered off the supplementary nutrition but will remain on the study and will follow the same schedule of events as those infants who continue study treatment, except where parents have not consented for continuation of data collection.

7.9 Compliance

Regular in-service education will be held for food services, nursing and medical staff, regarding the preparation and administration of the study nutrition.

A research nurse will monitor fluid balance charts to monitor adherence to the study protocol during the intervention phase, including use of infant formula outside of the study nutrition. Where non-compliance is detected, the number of non-compliant feeds (e.g. participant receive supplementary nutrition other than randomised group) will be recorded on the case record.

7.10 Outcome measures

Primary: Time to full enteral feeds (days), defined as 150ml/kg/d.

Secondary:

- Feeding complications, including frequency of feed intolerance, defined as the number of episodes where there was a medical decision to stop or reduce oral feeds, time to independent feeding (full suck feeds), duration of use of IV glucose, duration of use of parenteral nutrition and IV lipids, frequency and duration of use of other nutritional supplements (e.g. pentavite).
- Growth parameters, including time to regain birth weight, weight z-score for gestational age and change in weight z-score from birth to initial discharge home using Fenton growth charts, length, weight and head circumference at discharge, 2, and 4 months' CA.
- Neonatal complications including proven late onset sepsis, proven necrotising enterocolitis.
- Length of initial hospital stay, frequency and length of any readmissions up to 6 months' CA
- Body composition, including fat free mass as a % of total body mass at discharge, 2 and 4 months' CA assessed using air displacement plethysmography
- Breast milk feeds (fully or any) and method of feed (via bottle, at breast) at discharge, 2, 4 and 6 months' CA based on maternal self-report

7.11 Data collection

7.11.1 At enrolment

A research nurse/assistant will record the following baseline characteristics from the maternal and infant case notes and baseline questionnaire records: Maternal age at delivery, height and weight, ethnicity, education, occupation, parity, smoking status, mode of delivery, and medical and obstetric complications (e.g. gestational diabetes), use of antenatal corticosteroids, and factors contributing to preterm birth (e.g. chorioamnionitis) will be ascertained from review of maternal medical records. For infants, birth weight, length and head circumference, Apgar score at 5 and 10 minutes, and use of parenteral nutrition and IV fluid prior to enrolment, as well as day and type of first enteral feed. Maternal self-reported breastfeeding history and feeding intention for this infant (breastfeeding, formula, mixed feeding or undecided) will be assessed by completing a questionnaire at the time of enrolment.

A record of the total number of infants screened but not enrolled will also be maintained.

7.11.2 During intervention period

As part of routine care, the volume of all fluids (enteral or parenteral) an infant receives, along with number of breastfeeds is recorded in real time in a fluid balance chart kept in the infant medical record. All treating clinicians will be blinded to the study allocation and will be asked to record fluid intake as millilitres of ‘study nutrition’. This information will be extracted from the case notes by the research nurse/assistant to permit calculation of the total millilitres of either donor milk or formula given in the intervention period. Daily weight will be undertaken by the neonatal unit staff as per standard care. Feed tolerance and compliance with study nutrition allocation will be checked daily by the research nurse. Weight, length and head circumference will be measured on day 1 and day 8 (or discharge, if earlier) by the research nurse. Date of maternal and neonatal discharge home will be recorded by the research nurse.

7.11.3 At discharge and study follow-up assessments

At initial discharge and when the infant is 2 months’ and 4 months’ CA, all families will be asked to attend a study assessment on site to assess growth, body composition, and breastfeeding and infant health outcomes. Infant weight, length and head circumference will be measured according to standardised procedures. Body composition will be assessed using air displacement plethysmography, where infants are placed in a PEA POD (COSMED, Concord, CA, USA). This assessment will be undertaken by the research nurse

and is anticipated to take between 30 minutes to 1 hour. Maternal-completed questionnaires will assess current mode of feeding and ascertain any infant hospital readmissions.

In the event that an onsite assessment is not permissible (e.g. due to restrictions around COVID-19), a modified study assessment designed to collect information about growth and maternal questionnaires will be sent via text message to parents. The assessment links will be generated using the REDCap database platform.

An additional questionnaire will be sent to families at 6 months' CA to assess mode of feeding and infant readmissions.

7.11.4 Use of linked administrative data

To counteract COVID-19-related delays in undertaking follow up appointments or the possibility of having to cease follow up visits all together (e.g. due to high levels of community transmission after the borders open up), information relevant to the trial outcomes will also be sought from state-based government data collections about hospital separations and emergency department presentations as well as outpatient visits. The trial patient information sheet and consent form have been modified to include information about access to information held in these datasets.

The relevant datasets in South Australia include: SA Public Hospital Separations, SA Public Hospital Emergency Department and SA WCHN Child Health Record. The relevant datasets in Queensland include: Queensland Hospital Admitted Patient Data Collection and Queensland Emergency Department Collection.

To obtain this information data linkage agencies in South Australia (SA-NT DataLink) and Queensland (Data Linkage Queensland) will be supplied with identifying information about each participant (e.g. name, date of birth) and the GIFT study ID, and they will match these names with names that appear in the relevant datasets and then assign each participant a unique ID (a master linkage key). Following this, identifying information will be removed and the unique IDs will be sent to the data custodians of each dataset and merged with records for each participant. This creates a de-identified dataset which will be provided back to the trial statistician who will analyse the data on the basis of the GIFT study ID and treatment group allocation.

7.11.5 Assessment of lactation support and overall breastfeeding rates

As rates of maternal breastfeeding rates are strongly influenced by the level of lactation support in a neonatal unit, we will assess any changes to lactation support during the trial implementation period. This will involve collecting de-identified information about the number and allocation of qualified lactation consultations in each site, and requesting de-identified, routinely collected data on breastfeeding rates on discharge among infants born moderate to late preterm. We will compare the overall proportion of infants that were breastfeeding on discharge in the year preceding trial implementation with the rate at the completion of the trial.

7.12 Data management

Completed case report forms will be checked for completeness and accuracy by research nurses against the source data. Regular blinded reviews of data quality and data checks will be undertaken. Case report forms will be entered into a REDCap database, which uses a MySQL database via a secure web interface with data checks used during data entry to ensure data quality. REDCap includes a complete suite of features to support Health Insurance Portability and Accountability Act of 1996 (HIPAA) compliance, including a full audit trail, user-based privileges, and integration with the institutional LDAP server. The MySQL database, the web server and the deidentified linked dataset will be housed on secure servers operated by the South Australian Institute of Health and Medical Research (SAHMRI). The servers are in a physically secure location and are backed up nightly.

Electronic data storage is on a SAHMRI computer for at least 15 years after the completion of the study. The data will be stored on a restricted area of a file server; access to the files will be password protected and restricted to study personnel or authorised researchers from the study investigators' departments. The data will be deleted 30 years after it was last accessed for this study or other affiliated studies.

7.13 Sample size

A sample size of 100 infants per group will provide over 80% power to detect a reduction in the mean time to full enteral feeds (days) between the treatment groups of 0.5 standard deviations, with a two-sided alpha of 0.05 and 5% loss to follow-up. This calculation assumes 24% of infants will be twins and allows for a worst-case scenario of perfect correlation between outcomes of twins. This sample size exceeds the minimum

recommendations for pilot studies to provide accurate parameter estimates for planning a larger definitive trial (for further information see Teare et al, Trials 2014; 15:264).

7.14 Statistical methods

The primary outcome of time to full enteral feeds will be analysed using linear regression; no censoring is expected due to the length of the follow-up period. Generalised estimating equations (GEEs) will be used to account for clustering due to multiple births. Analyses will be performed on an intention-to-treat basis and a two-sided P-value <0.05 will be considered statistically significant. Secondary outcomes will be analysed using linear regression models for continuous outcomes and log binomial regression models for binary outcomes, with adjustment for clustering using GEEs. A detailed statistical analysis plan will be developed prior to the commencement of any analyses in consultation with the trial statistician, Dr Lisa Yelland.

7.15 Data and safety monitoring

The trial will be overseen by a Steering Committee, consisting of the study investigators. The committee will meet monthly to monitor recruitment progress, monitor protocol violations, plan donor milk supply needs, and advise on strategies to maximise recruitment and follow-up at each centre. To manage potential conflicts of interest, this committee will not have any direct role in data collection or analysis.

As this is a pilot study, an interim analysis will not be performed. An independent, blinded Serious Adverse Events (SAE) Committee comprising of a neonatal paediatrician and pathologist will meet every 6 months (or as required) to review adverse events. There are no adverse anticipated to be a unique consequence of participation in this trial, however, any infant deaths will be reviewed. The committee will determine whether there is any likelihood that the SAE is due to the intervention and provide reports to the relevant Human Research Ethics Committees (HREC).

Any event that may be of immediate or potential concern for an infant’s health or well-being will be reported immediately to the Principal Investigator. The Principal Investigator and clinical Co-Investigators will investigate all Adverse Events or Serious Advent Events, and provide additional information to the attending clinical team for them to assess and manage

the adverse event. The Principal Investigator (or a Co-Investigator) will report all adverse events to the HREC.

Harms associated with the intervention are unlikely, as all donor human milk will undergo microbiological screening before and after Holder pasteurisation (30 minutes at 62.5°C) according to Lifeblood Milk policies. All milk donors are screened in line with international milk banking to minimise risk of infectious disease transmission. Harms associated with the control group are unlikely as the trial will utilise infant formula that is current standard care.

7.16 Ethics, recruitment and dissemination

This Study Protocol, the Participant Information and the Participant Consent document and any subsequent modifications will be reviewed and approved by the Women's and Children's HREC and the Royal Brisbane & Women's HRE. A letter of protocol approval by HREC will be obtained prior to the commencement of the study, as well as approval for other study documents subject to HREC review.

7.16.1 Modifications of the protocol

This study will be conducted in compliance with the current version of the protocol. Any change to the Protocol document, Participant Information or Participant Consent form that affects the scientific intent, study design, patient safety, or may affect a participant's willingness to continue participation in the study is considered an amendment, and therefore will be written and filed as an amendment. All such amendments will be submitted to the HREC for approval prior to becoming effective.

7.16.2 Protocol deviations

All protocol deviations must be recorded in the patient medical record and on the case report form and must be reported to the Principal Investigator or Co-Investigators. Protocol deviations will be assessed for significance by the Principal Investigator (or Co-Investigators). Those deviations deemed to have a potential impact on the integrity of the study results, patient safety or the ethical acceptability of the trial will be reported to the HREC.

7.16.3 Consent

Nursing staff on the antenatal/gynae ward and neonatal unit will undertake the initial screen for potentially eligible infants and alert the research nurse/assistant of mothers/families to approach. Mothers who may be approached antenatally include those in preterm labour or who have a planned delivery at 32-36 weeks. Recruitment strategies will include posters and pamphlets placed in the neonatal unit and the postnatal ward advertising the study and providing information to potential families. The research nurse will approach parent/s of eligible infants either antenatally or as soon as possible after birth when the infant is clinically stable. Information regarding the study will be provided to the potential participants. The Participant Information form will describe the purpose of the study, the procedures to be followed, and the risks and benefits of participation.

The research nurse will conduct the informed consent discussion and will check that information provided is understood and answer any questions about the study. Consent will be voluntary and free from coercion. If the Co-Investigators are discussing the study or gaining consent from the participant's parents whilst also acting in a clinical capacity for that infant, the participants will be given enough time to consider the information provided and will have the opportunity to submit the signed Participant Consent form and their contact details to a third party (such as Administration Officer, nursing Shift Coordinator from the Special Care Baby Unit or Neonatal Intensive Care Unit, which ever unit the baby is not admitted to). A copy of the Participant Consent form and the Participant Information form will be given to the parents and also documented in the infant's medical record and study Case Report Form.

7.16.4 Confidentiality

Participant confidentiality is strictly held in trust by the participating investigators and research staff and their agents. The Study Protocol, documentation, data and all other information generated will be held in strict confidence. No information concerning the study or the data will be released to any unauthorised third party, without prior written approval of the Principal Investigator (or Co-Investigators). Regulatory authorities may inspect all documents and records required to be maintained by the Investigators, including but not limited to, medical records for the infants in this study. The clinical study site will permit

access to such records. Clinical information will not be released without written permission of the parents, except as necessary for monitoring by HREC or regulatory agencies.

7.16.5 Use of data and publication policy

Results of the research study will be disseminated to participants through the provision of results of the study posted or emailed to participants at the study conclusion. It is anticipated that the findings will be presented at professional meetings and written up for publication in peer review journals.

8. REFERENCES

1. Hay WW. Nutritional Support Strategies for the Preterm Infant in the Neonatal Intensive Care Unit. *Pediatric Gastroenterology, Hepatology & Nutrition.* 2018;21(4):234.
2. Australian Institute of Health and Welfare. Australia's mothers and babies 2017—in brief. *Perinatal statistics series no 35 Cat no PER 100 Canberra: AIHW.* 2019.
3. Gouyon JB, Iacobelli S, Ferdynus C, Bonsante F. Neonatal problems of late and moderate preterm infants. 2012;17(3):146-152.
4. Escobar GJ, Clark RH, Greene JD. Short-Term Outcomes of Infants Born at 35 and 36 Weeks Gestation: We Need to Ask More Questions. *Seminars in perinatology.* 2006;30(1):28-33.
5. Lapillonne A, Bronsky J, Campoy C, et al. Feeding the Late and Moderately Preterm Infant: A Position Paper of the European Society for Paediatric Gastroenterology, Hepatology and Nutrition Committee on Nutrition. *Journal of pediatric gastroenterology and nutrition.* 2019;69(2):259-270.
6. Khan K, Petrou S, Dritsaki M, et al. Economic costs associated with moderate and late preterm birth: a prospective population-based study. *BJOG: An International Journal of Obstetrics & Gynaecology.* 2015;122(11):1495-1505.
7. Asadi S, Bloomfield FH, Harding JE. Nutrition in late preterm infants. *Seminars in perinatology.* 2019;43(7):151160.
8. Alexander T, Bloomfield FH. Nutritional management of moderate-late preterm infants: Survey of current practice. *Journal of paediatrics and child health.* 2019;55(3):338-342.
9. Austin S, De Castro CA, Sprenger N, et al. Human Milk Oligosaccharides in the Milk of Mothers Delivering Term versus Preterm Infants. *Nutrients.* 2019;11(6):1282.
10. Chantry CJ, et al. In-hospital formula use increases early breastfeeding cessation among first-time mothers intending to exclusively breastfeed J Pediatr. 2014 Jun;164(6):1339-45.e5.
11. Tully KP, et al. The Relationship Between Infant Feeding Outcomes and Maternal Emotional Well-being Among Mothers of Late Preterm and Term Infants: A Secondary, Exploratory Analysis. Adv Neonatal Care. 2017 Feb;17(1):65-75
12. Quigley M, Embleton ND, McGuire W. Formula versus donor breast milk for feeding preterm or low birth weight infants. *Cochrane Database of Systematic Reviews.* 2019.
13. Johnson MJ, Wootton SA, Leaf AA, Jackson AA. Preterm Birth and Body Composition at Term Equivalent Age: A Systematic Review and Meta-analysis. *Pediatrics.* 2012;130(3):e640-e649.
14. Williams T, et al, Use of Donor Human Milk and Maternal Breastfeeding Rates: A Systematic Review. J Hum Lact. 2016 May;32(2):212-20.
15. Davies DP. Adequacy of expressed breast milk for early growth of preterm infants. *Archives of Disease in Childhood.* 1977;52(4):296-301.
16. Raiha NC, Heinonen K, Rassin DK, Gaull GE. Milk protein quantity and quality in low-birthweight infants: I. Metabolic responses and effects on growth. *Pediatrics.* 1976;57(5):659-684.
17. Schultz K, Soltész G, Mestyán J. The metabolic consequences of human milk and formula feeding in premature infants.. *Acta Paediatrica.* 1980;69(5):647-652.

9. APPENDIX 1

Nutrition support protocol

Inclusion: 32+0 and 36+6 completed weeks' gestation at birth; birth weight ≥ 1500g; admitted to the neonatal unit; clinically stable; ready to commence enteral feeds or commenced human milk feeds but with insufficient maternal breast milk and ≤ 4 days of age.

Exclusion: metabolic disorder that precludes breastfeeding; major congenital malformation either likely to interfere with the ability to ingest milk or requiring surgery in the first 6 months of life (e.g. complex congenital heart disease); given infant formula prior to randomisation.

Intervention: supplementary pasteurised donor human milk
Control: term infant formula

Duration of intervention: Cease on study day 8 (day supplementary feeds started + 7 days), or earlier if maternal expressed breast milk (EBM) supply is sufficient to feed at a rate of 150ml/kg/day or if discharged from the hospital.

**NUTRITION PROTOCOL**		
**Day of birth**	Commence nutrition protocol as per local unit standards, typically<br>▪ Commencement of enteral feeds with mother's own EBM as soon as possible after birth or when clinically stable, starting at 10-15 ml/kg/day, and increasing 10-15 ml/kg/day or as per clinician discretion.<br>▪ IV fluids for maintenance and/or IV glucose and electrolytes used as per unit standards	
**Decision made by treating team to commence supplementary enteral feeds**	Volume of mother's own EBM available is not sufficient for growth needs, e.g. weight gain <15g/kg/day or as determined by the treating team<br><br>INFANT IS ELIGIBLE FOR TRIAL IF: human milk feeds commenced by day 4, has not received any infant formula and meets all other eligibility criteria	
	**Randomisation**	
**INTERVENTION PHASE (8 days in total)**	**INTERVENTION NUTRITION**<br>**Pasteurised donor human milk**	**CONTROL NUTRITION**<br>**Term infant formula**
	▪ Commence at unit protocol for age (ml/kg/day) up to 160-180 ml/kg/day<br>▪ Advance feeds as per local protocols (e.g. at 10-30 ml/kg/day)	▪ Commence at unit protocol for age (ml/kg/day) up to 160-180 ml/kg/day<br>▪ Advance feeds as per local protocols (e.g. at 10-30 ml/kg/day)

	▪ Feed first with mother's own EBM, continue lactation support, encourage direct breast feeds where possible<br>▪ Decrease volume of supplementary nutrition as mother's own milk supply increases<br>▪ Cease when<br>- mother's own milk supports target fluid intake OR<br>- day 8 of supplementary feeds OR<br>- hospital discharge<br>▪ Do not give any supplementary infant formula	▪ Feed first with mother's own EBM, continue lactation support, encourage direct breast feeds where possible<br>▪ Decrease volume of supplementary nutrition as mother's own milk supply increases<br>▪ Cease when<br>- mother's own milk supports target fluid intake OR<br>- day 8 of supplementary feeds OR<br>- hospital discharge
	**If the decision to fortify is made**	
	▪ First fortify mother's own milk<br>▪ If insufficient volume of mother's own milk is available, fortify donor milk	▪ First fortify mother's own milk<br>▪ If insufficient volume of mother's own milk is available, switch to preterm formula
	**Fluid and nutrition monitoring in both groups during intervention phase**	
	▪ The rate of advancement of feeds is at the discretion of the treating team. Babies with growth restriction may require slower advancement of feeds.<br>▪ IV glucose and electrolytes to be used as per unit standards, no restrictions on medications or nutritional supplements (e.g. Pentavite) <u>with the exception of formula.</u>	
**POST-INTERVENTION PHASE IN HOSPITAL**	If the infant remains admitted at the end of the intervention phase and supplementary feeds are still required, infants will be fed according to local unit protocols.	
**STUDY FOLLOW UP**	Assessments and/or questionnaires at hospital discharge, 2, 4, and 6 months' CA	

Study investigations and anthropometric measurements

Assessment	Weight, length, head circumference	Body composition	Maternal questionnaires		
			Baseline characteristics	Feeding method	Infant readmissions
Day 1 of intervention period (standard care)	X		X		
Day 8 or date of discharge if earlier (standard care)	X				
Date of discharge (study visit)	X	X		X	
2 months' CA (study visit)	X	X		X	X
4 months' CA (study visit)	X	X		X	X
6 months' CA (study visit)				X	X
