# Supplementary material for: Supplemental Donor Milk vs Infant Formula in Moderate to Late Preterm Infants: A Randomized Clinical Trial
Source: JAMA Pediatr. 2025 Aug 4;179(10):1065–73. doi: 10.1001/jamapediatrics.2025.2365 (PMC12322819; doi:10.1001/jamapediatrics.2025.2365)
Supplement: Supplement 2. — Statistical Analysis Plan [file jamapediatr-e252365-s002.pdf]

Giving donor milk instead of formula in moderate-late preterm infants: the GIFT trial

Statistical Analysis Plan

Trial Registration ACTRN12621000529842

SAP Version 1.0, 8 December 2023

SAP Authors

Dr Lisa Yelland (Statistical Investigator)
Biostatistics Unit
South Australian Health & Medical Research Institute

Dr Alana Cuthbert (Trial Statistician)
Biostatistics Unit
South Australian Health & Medical Research Institute

Prof Alice Rumbold (Principal Investigator)
SAHMRI Women & Kids
South Australian Health & Medical Research Institute

Approved by

	Signature	Date
Lisa Yelland		11/12/2023
Alana Cuthbert		8/12/2023
Alice Rumbold		11/12/2023

Abbreviations

CA	corrected age
GA	gestational age
GEE	generalised estimating equation
PNS	neonatal parenteral nutrition solution

Table of Contents

1. INTRODUCTION 3
Background 3
Objectives 3
Estimand for primary objective 3
2. STUDY METHODS 3
Trial Design 3
Participants 3
Treatments 3
Blinding 3
Randomisation 3
Sample size 4
Further details 4
3. GENERAL STATISTICAL ISSUES 4
Framework 4
Confidence intervals and p-values 4
Analysis population 4
Covariate adjustment 4
Clustering 4
Subgroup analyses 5
Missing data 5
Outliers 5
Timing of analyses 5
4. DESCRIPTIVE STATISTICS 5
Flow diagram 5
Adherence and protocol deviations 5
Baseline characteristics 6
Breastfeeding support summary 7
Missing data 8
Blinding assessment 9
5. STATISTICAL ANALYSIS 9
Primary outcome 9
Secondary outcomes 9
Descriptive outcomes 11
6. REFERENCES 12

1. INTRODUCTION

Background

Poor early nutrition is hypothesised to contribute to many of the short- and long-term complications seen in moderate and late preterm infants. Supplementary formula is often required, due to insufficient maternal milk supply, but can lead to feed intolerance and other problems. Donor human milk is a promising alternative that is currently prioritised for use in very preterm infants (<32 weeks' gestation) and those with a birth weight <1500g.

Objectives

To compare the effect of supplementary pasteurised donor human milk versus standard infant formula on the time to establish full enteral feeds (primary objective), feed intolerance, growth, breastfeeding and length of stay (secondary objectives) in clinically well preterm infants born between 32+0 and 36+6 weeks' gestation with a birth weight ≥1500g.

Estimand for primary objective

In clinically well preterm infants born between 32+0 and 36+6 weeks' gestation with a birth weight ≥1500g who survive to reach full enteral feeds, what is the difference in the mean time in days to establish full enteral feeds between donor human milk versus standard infant formula, regardless of any non-adherence to the study protocol, the amount of supplementary feeding required, or loss of access to the study milk during the intervention phase (e.g. due to hospital discharge, transfer to a different hospital or participation in the neonatal early discharge program).

2. STUDY METHODS

Trial Design

Multicentre, blinded, parallel design, pilot randomised controlled trial.

Participants

Infants born between 32+0 and 36+6 completed weeks' gestation; birth weight ≥ 1500g; admitted to the neonatal unit; clinically stable; ready to commence enteral feeds or commenced enteral feeds but with insufficient maternal breast milk; and ≤ 4 days old.

Treatments

Pasteurised donor human milk (intervention) versus standard term formula (control) for up to 8 days as a supplement to maternal breast milk when required.

Blinding

Participants, families, clinical teams and research staff, including research nurses, outcome assessors and data analysts, are blinded to the randomisation groups. Study milk is provided in colour-coded syringes/bottles with 2 colours per treatment group to reduce the risk of unblinding.

Randomisation

Infants are randomly allocated to the intervention or control group in a 1:1 ratio using REDCap, with cluster randomisation of infants from the same birth. The randomisation is stratified by centre (WCH, RBWH) and GA (32+0 to 33+6, 34+0 to 36+6) and uses randomly permuted blocks of varying size to assign infants to one of the 4 colour groups (2 colours per treatment group). As RBWH does not treat infants born <34 weeks, there are 3 strata rather than 4.

Sample size

A sample size of 100 infants per group provides over 80% power to detect a reduction in the mean time to full enteral feeds (days) between the treatment groups of 0.5 standard deviations, with a two-sided alpha of 0.05 and 5% loss to follow-up. This calculation assumes 24% of infants will be twins and allows for a worst-case scenario of perfect correlation between outcomes of twins.

Further details

Refer to the current version of the trial protocol (version 1.3, 25th November 2021) for further details on the study design.

3. GENERAL STATISTICAL ISSUES

Framework

All comparisons will be undertaken assuming a standard superiority hypothesis testing framework.

Confidence intervals and p-values

For each outcome, a 95% confidence interval will be reported to express uncertainty about the estimated treatment effect, with the effect taken to be statistically significant if the p-value for the two-sided comparative test is <0.05. No multiplicity adjustment will be made for the number of hypothesis tests performed for secondary outcomes, as these are of less importance than the primary outcome.

Analysis population

The analysis population will generally include all randomised participants in their randomised treatment group (intention-to-treat approach), regardless of adherence or any protocol deviations. Where the death of a participant means the outcome cannot be measured, the analysis population will be restricted to survivors to the relevant time point or event.

Covariate adjustment

Analyses will be adjusted for the two stratification variables: centre (WCH, RBWH) and GA (32+0 to 33+6, 34+0 to 36+6), since this is the simplest correct analysis following stratified randomisation(1). Adjustment will also be made for multiple birth (yes/no). No other covariate adjustment is planned, however supplementary analyses may be performed for the primary outcome with adjustment for additional covariates if substantial imbalance is identified in descriptive analyses of key baseline covariates (see Section 4) for the purpose of exploring the impact on the primary trial results. For binary outcomes with insufficient events to support a sensible fully adjusted analysis, adjustment covariates will be sequentially removed (multiple birth, then site, then GA), or an unadjusted analysis may be performed.

Clustering

Infants from both single and multiple births are eligible to participate and outcomes of infants from the same birth are expected to be correlated, resulting in partially clustered data. Clustering due to multiple births will be taken into account in the analysis of all infant-level outcomes measured at a single time point using the generalised estimating equation (GEE) method of estimation. An independence working correlation structure will be specified to produce treatment effect estimates with an infant-level interpretation(2). For outcomes measured repeatedly over time, GEEs will be fit with an interaction between treatment and time point and be used to estimate the treatment effect at each time point. Clustering will be accounted for at the mother level, as when clustering is nested within GEEs (time points within infants, infants within mothers), it is only necessary to account for the highest level of clustering(3).

Subgroup analyses

There are no planned subgroup analyses due to the relatively small sample size.

Missing data

All analyses will be performed on the available data (complete case analysis). Very little missing data is expected on the primary outcome.

Outliers

Outliers will be queried during data collection and cleaning. Unless confirmed as a data entry error, outliers will not be excluded from the primary analyses. Supplementary analyses may be undertaken excluding participants with extreme values, where present, on a given outcome to assess their impact on the results.

Timing of analyses

The database will be locked for analysis once data collection and cleaning are complete and this statistical analysis plan has been approved. Blinded treatment codes (e.g. 'A' and 'B') will then be made available for analysis, with the blinding to be broken following review of the blinded results by the principal investigator.

4. DESCRIPTIVE STATISTICS

Flow diagram

Information will be presented by treatment group (where appropriate) to allow completion of the flow diagram, including number of participants:

- Screened
- Eligible to participate
- Ineligible to participate (by reason)
- Providing informed consent
- Not providing informed consent (by reason)
- Randomised
- Withdrawn
- Lost to follow-up
- With primary outcome data available

Adherence and protocol deviations

Descriptive statistics will be presented by treatment group on

Measures of study milk intake

- Any use of study milk
- Number of days using study milk
- Total volume of study milk given *(mL)*
- Volume of study milk given as a percentage of total milk intake *(%)*
- Method of feeding study milk
  - Bottle *(yes, no)*
  - Syringe *(yes, no)*
  - Gavage *(yes, no)*
  - Perfusor *(yes, no)*
  - Other method *(yes, no)*
- Study day at first study milk intake

- Use of human milk fortifier *(yes, no)*
- Extra calories ordered *(yes, no)*
- Use of protein additive *(yes, no)*
- Use of polyjoule *(yes, no)*
- Use of other supplements *(yes, no)*
- Cessation of study milk for reasons other than 'completed'

Protocol deviations

- Ineligible participant randomised
- Randomised in the wrong stratum
- Received the wrong study milk
- Use of non-protocol formula
- Use of study milk outside intervention period
- Use of study milk when mother's own milk was available
- Unblinding

For the primary outcome and select secondary outcomes, a supplementary analysis excluding any ineligible participants randomised may be performed.

Baseline characteristics

Descriptive statistics will be presented by treatment group on the following mother level and infant level characteristics:

Mother level characteristics

- Mother's age at delivery *(years)*
- Mother's BMI *(weight kg/(height m)^2)*
- Parity *(1, 2, 3, 4, 5 or more)*
- Gestational diabetes *(yes, no)*
- Pre-existing diabetes *(yes, no)*
- Use of antenatal corticosteroids *(yes, no)*
- Country of birth *(Australia, Oceania, Northeast Asia, Southeast Asia, Southern and Central Asia, Northwest Europe, Southern and Eastern Europe, Americas, North Africa and Middle East)*
- Aboriginal/Torres Strait Islander status *(Aboriginal, Torres Strait Islander, Both Aboriginal and Torres Strait Islander, Neither Aboriginal or Torres Strait Islander)*
- Highest level of education *(masters or doctorate, undergraduate or honours degree, diploma, certificate/apprenticeship, secondary school, did not finish secondary school)*
- In paid workforce in last 12 months *(yes, no)*
- Occupation *(Clerical and administrative worker, community and personal service worker, labourer, manager, professional, sales worker, technicians and trade workers, other)*
- Index of Relative Social Disadvantage quintile *(1, 2, 3, 4, 5)*
- Smoked cigarettes during pregnancy *(yes, no)*
- Currently smoking cigarettes *(yes, no)*
- Drank alcohol during pregnancy *(yes, no)*
- Consumed alcohol since giving birth *(yes, no)*
- Maternal allergic disease:
    - Food allergy *(yes, no)*
    - Asthma *(yes, no)*
    - Eczema *(yes, no)*
    - Hayfever *(yes, no)*
    - Pet allergy *(yes, no)*

Infant level characteristics

- Age at randomisation *(days)*
- Sex *(male, female)*
- Plurality *(singleton, twin, triplet)*
- Mode of delivery *(spontaneous vaginal birth, induction leading to vaginal birth, caesarean (planned), caesarean (emergency))*
- Factors contributing to preterm birth
    - Chorioamnionitis *(yes, no)*
    - Pre-eclampsia *(yes, no)*
    - Placental abruption *(yes, no)*
    - Poor foetal growth *(yes, no)*
    - Foetal distress *(yes, no)*
    - Other *(yes, no)*
- Apgar score at 1 minute *(0-3, 4-6, 7-10)*
- Apgar score at 5 minutes *(0-3, 4-6, 7-10)*
- Birth weight *(g)*
- Birth length *(cm)*
- Birth head circumference *(cm)*
- Age in days at first milk feed *(days)*
- Type of first milk feed *(maternal EBM, study milk, formula)*
- Method of first milk feed *(breastfeeding (direct), bottle, gavage feeding, finger-feeding, syringe, other)*
- Number of days infant had any breast milk prior to randomisation
- Hypoglycaemia prior to randomisation *(yes, no)*
- Use of IV dextrose prior to randomisation *(yes, no)*
- Use of dextrose gel or oral dextrose prior to randomisation *(yes, no)*
- Use of PNS prior to randomisation *(yes, no)*
- Family history of allergic disease (includes maternal, paternal, or sibling):
    - Food allergy *(yes, no)*
    - Asthma *(yes, no)*
    - Eczema *(yes, no)*
    - Hayfever *(yes, no)*
    - Pet allergy *(yes, no)*

Breastfeeding support summary

Descriptive statistics will be presented by treatment group on measures of breastfeeding support recorded at baseline, discharge, 2 months CA, 4 months CA, and 6 months CA. Measures will be summarised at the mother level or infant level, as appropriate.

Baseline visit

- Have you ever breastfed before? *(yes, no, N/A (first baby))*
- Before giving birth, how did you intend to feed this baby? *(breast milk only, breast milk and formula, formula only, undecided, other)*
- Did you attend any classes whilst pregnant that included breastfeeding information? *(yes, no)*
- Reason breastfeeding classes not attended
- Since giving birth, how long do you intend to give this baby breast milk? *(less than one month, 1-6 months, 7-12 months, 13-24 months, as long as I can, unsure/undecided)*
- How confident do you feel about breastfeeding this baby for as long as planned? *(very confident, confident, uncertain, very uncertain, don't know/unsure)*
- Since giving birth to this baby, have you had access to breastfeeding support? *(yes, no)*

- Are you currently expressing breastmilk? *(yes, no)*
- Number of times expressing per day *(1-5, 6-10, 11 or more, other)*

Study visit 1

- Since giving birth, have you taken any medications to boost your breast milk supply? *(yes, no)*
    - Domperidone *(yes, no)*
    - Metoclopramide *(yes, no)*
    - Other *(yes, no)*
- Since giving birth, have you taken any supplements, foods or ingredients to try and boost your breast milk supply? *(yes, no)*
    - Fenugreek *(yes, no)*
    - Brewer's yeast *(yes, no)*
    - Lactation cookies *(yes, no)*
    - Ginger *(yes, no)*
    - Other *(yes, no)*
- Overall, after giving birth to your baby, do you feel that the hospital provided you with enough support with breastfeeding? *(yes, no, unsure)*
- How are you currently feeding your baby? *(breast milk only, at the breast, breast milk only, via bottle or tube, breast milk only, feeding at the breast AND via bottle or tube, mainly breast milk with some formula, an equal mix of breast milk and formula, mainly formula with some breast milk, formula only (no breast milk), other)*
- How long do you intend to give this baby breast milk? *(0-6 months, 7-12 months, 13-24 months, as long as I can, unsure/undecided, other)*
- How confident do you feel about breastfeeding this baby for as long as planned? *(very confident, confident, uncertain, very uncertain, don't know)*

2- and 4-month CA visit

- How are you currently feeding your baby? *(breast milk only, at the breast, breast milk only, via bottle or tube, breast milk only, feeding at the breast AND via bottle or tube, mainly breast milk with some formula, an equal mix of breast milk and formula, mainly formula with some breast milk, formula only (no breast milk), other)*
- How long do you intend to give this baby breast milk? *(0-6 months, 7-12 months, 13-24 months, as long as I can, unsure/undecided, other)*
- How confident do you feel about breastfeeding this baby for as long as planned? *(very confident, confident, uncertain, very uncertain, don't know)*
- Have you started feeding your baby any solid or semi-solid food? *(yes, no)*

6-month CA visit

- How are you currently feeding your baby? *(breast milk only, at the breast, breast milk only, via bottle or tube, breast milk only, feeding at the breast AND via bottle or tube, mainly breast milk with some formula, an equal mix of breast milk and formula, mainly formula with some breast milk, formula only (no breast milk), other)*
- Overall, are you happy with the length of time you have been able to breastfeed or express breast milk for your baby? *(Yes - I am still giving breast milk to my baby, yes - it was as long as I planned, yes it was longer than I planned, no - I originally planned to breastfeed for longer, not sure - I have mixed feelings, not applicable - I never intended to breastfeed, other)*

Missing data

The number and percentage of missing values will be presented by treatment group for all baseline characteristics, measures of adherence, measures of breastfeeding support and outcome variables.

Blinding assessment

The number and percentage of mothers who guessed their infant(s) were in the donor human milk group, standard term formula group or unsure will be reported by treatment group at the mother level. The blinding index of Bang et al.(4) will also be reported by treatment group.

5. STATISTICAL ANALYSIS

Primary outcome

Time in days from randomisation until full enteral feeds, defined as 150ml/kg/d.

The primary outcome will be calculated as the date when full enteral feeds were reached minus the date of randomisation.

The analysis of the primary outcome will be performed on the available data using linear regression, as no censoring is expected due to the length of the follow-up period. Robust variance estimation will be used due to the likely skewed distribution of the data. GEEs with an independence working correlation structure will be used to account for clustering of infants within mothers due to the inclusion of multiple births, and adjustment will be made for centre, GA, and multiple birth. Results will be presented as a difference in the mean number of days (donor human milk minus standard term formula) with a 95% confidence interval and 2-sided p-value.

Secondary outcomes

Secondary outcomes will be compared between treatment groups as outlined in the table below and following the general statistical approaches described in Section 3.

	Outcome Definition	Analysis Method	Notes
	Feeding Complications		
**1**	**Primary outcome:**<br>**Time in days from randomisation until full enteral feeds reached**	Linear GEE with robust variance estimation to estimate difference in means (as described above)	
2	Number of episodes of feed intolerance, defined by a documented decision to delay or stop enteral feeds, during the intervention phase	Negative binomial GEE with offset for number of days of study milk to estimate rate ratio	
3	Number of episodes of feed intolerance, defined by a documented decision to delay or stop enteral feeds, from randomisation until hospital discharge	Negative binomial GEE with offset for number of days between randomisation and discharge to estimate rate ratio	
4	Time in days from randomisation until full suck feeds is reached,	Cox proportional hazards model with a cluster adjusted variance estimator to estimate hazard ratio	Time to event variable will be censored at either the date of hospital discharge, or the date of discharge from NED, depending on discharge type.
5	Duration of use of IV glucose in days, from randomisation until hospital discharge	Linear GEE with robust variance estimation to estimate difference in means	
6	Duration of use of parenteral nutrition in days, from randomisation	Linear GEE with robust variance estimation to estimate difference in means	

	until hospital discharge		
	Growth parameters		
7	Time in days from randomisation until birth weight is regained	Cox proportional hazards model with a cluster adjusted variance estimator to estimate hazard ratio	Time to event variable will be censored at either the date of hospital discharge, or the date of discharge from NED, depending on discharge type.
8	Weight z-score for gestational age, calculated using Fenton growth charts(5) and assessed at hospital discharge	Linear GEE to estimate difference in means	Fenton z-score at discharge calculated using weight, gestational age (see below) and sex<br><br>If infant weight at discharge is not available, then the weight measured on the final day of feeding assessment will be used instead.
9	Change in weight z-score for gestational age, calculated using Fenton growth charts and assessed using the weight measurements closest but prior to randomisation and at hospital discharge	Linear GEE to estimate difference in means	(Fenton z-score at discharge) – (Fenton z-score at randomisation)
10	Weight (kg) at<br>• hospital discharge<br>• 2 months CA<br>• 4 months CA	Linear GEE with treatment group, time, their interaction, and variables for adjustment included in the model, and clustering on the mother to estimate differences in means at each timepoint separately.	Where infant weight has been measured multiple times at the same visit (as specified in study protocol):<br>• If 2 measurements were taken, the mean will be taken as the infant weight.<br>• If 3 measurements taken, the median will be taken as the infant weight.<br><br>If infant weight at discharge is not available, then the weight measured on the final day of feeding assessment will be used instead.
11	Length (cm)<br>• hospital discharge<br>• 2 months CA<br>• 4 months CA	As for weight	As for weight
12	Head circumference (cm)<br>• hospital discharge<br>• 2 months CA<br>• 4 months CA	As for weight	As for weight
13	Fat free mass (%) at<br>• hospital discharge<br>• 2 months CA<br>• 4 months CA	As for weight	
	Neonatal complications		

14	Confirmed sepsis, assessed from randomisation until hospital discharge	Log binomial GEE to estimate relative risk	
15	Confirmed necrotising enterocolitis, assessed from randomisation until hospital discharge	Log binomial GEE to estimate relative risk	
	Hospitalisations		
16	Time in days from randomisation until initial hospital discharge, including time spent in the neonatal early discharge program	Linear GEE with robust variance estimation to estimate difference in means	
17	Time in days from randomisation until initial hospital discharge, excluding time spent in the neonatal early discharge program	Linear GEE with robust variance estimation to estimate difference in means	
18	Number of hospital readmissions, assessed from initial hospital discharge until 6 months CA	Negative binomial GEE to estimate ratio of means	Sum of reported number of hospital admissions recorded at the 2, 4 and 6 month appointments.<br>This outcome is only reported for participants with a response recorded at all 3 appointments.<br>Note due to structure of survey questions, at most one visit is assumed to have occurred between the 4 and 6 month appointment.
19	Total length of readmissions in days, assessed from initial hospital discharge until 6 months CA	Linear GEE with robust variance estimation to estimate difference in means	As above, with sum of reported number of days spent in hospital.<br>Only reported for participants with a response recorded at all 3 appointments.
	Breast milk intake		
20	Exclusive breast milk feeding at<br>• hospital discharge<br>• 2 months CA<br>• 4 months CA<br>• 6 months CA	Log binomial GEE with treatment group, time, their interaction, and variables for adjustment included in the model, with clustering on the mother to estimate relative risks at hospital discharge, 2 months CA, and 4 months CA separately. Due to low anticipated numbers of exclusively breastfed infants at 6 months, this time point will be summarised descriptively only.	Responses indicate that infant is fed breast milk only at discharge, 2, 4 and 6 month CA appointments<br>Note if infant has had any liquids other than water, or has started solids, then they are no longer considered to be exclusively breast milk feeding.
21	Any breast milk feeding at<br>• hospital discharge<br>• 2 months CA<br>• 4 months CA<br>• 6 months CA	As for exclusive breast feeding, except that outcomes at 6 months will be included in the analysis.	

Descriptive outcomes

The following outcomes will be summarised descriptively

- Number of episodes of vomiting during the intervention phase (total)
- Number of days where vomit with green aspirate was present during the intervention phase
- Number of episodes of hypoglycaemia during the intervention phase
- Use of supplementary feeds after study nutrition ceased (yes, no)
- For infants who required supplementary feeds after study nutrition ceased, the duration of use of supplementary feeds. For infants who were discharged on mixed feeding (and therefore have unknown duration of supplementary feeds), the duration of supplementary feeds while in hospital will be reported.
- Number of episodes of hypoglycaemia after study nutrition ceased
- Confirmed spontaneous intestinal perforation not NEC–associated
- Jaundice requiring phototherapy from randomisation until hospital discharge
- Main respiratory diagnosis:
    - Normal (no respiratory support) *(yes, no)*
    - Non-specific *(yes, no)*
    - Hyaline membrane disease *(yes, no)*
    - Meconium aspiration *(yes, no)*
    - Pneumonia *(yes, no)*
    - Persistent pulmonary hypertension *(yes, no)*
    - Apnoea *(yes, no)*
    - Congenital malformation *(yes, no)*
    - Peri surgical *(yes, no)*
    - Newborn encephalopathy/Hypoxic ischaemic encephalopathy *(yes, no)*
    - Other *(yes, no)*
- Fat mass in grams
    - At initial visit after discharge
    - At 2 months CA
    - At 4 months CA

6. REFERENCES

1. Kahan BC, Morris TP. Improper analysis of trials randomised using stratified blocks or minimisation. Statistics in Medicine. 2012 Feb 20;31(4):328–40.

2. Yelland LN, Sullivan TR, Pavlou M, Seaman SR. Analysis of Randomised Trials Including Multiple Births When Birth Size Is Informative. Paediatric and perinatal epidemiology. 2015 Nov 1;29(6):567.

3. Miglioretti DL, Heagerty PJ. Marginal Modeling of Nonnested Multilevel Data using Standard Software. American Journal of Epidemiology. 2006 Nov 16;165(4):453–63.

4. Bang H, Ni L, Davis CE. Assessment of blinding in clinical trials. Control Clin Trials. 2004 Apr;25(2):143–56.

5. Fenton TR, Kim JH. A systematic review and meta-analysis to revise the Fenton growth chart for preterm infants. BMC Pediatrics. 2013 Apr 20;13(1):59.
