# Supplementary material for: Supplemental Donor Milk vs Infant Formula in Moderate to Late Preterm Infants: A Randomized Clinical Trial
Source: JAMA Pediatr. 2025 Aug 4;179(10):1065–73. doi: 10.1001/jamapediatrics.2025.2365 (PMC12322819; doi:10.1001/jamapediatrics.2025.2365)
Supplement: Supplement 3. — eTable 1. Which Group Mothers Thought Their Baby Was In, by Treatment Group eAppendix 1. Sensitivity Analyses for the Primary Outcome eTable 2. Time to Full Enteral Feeds, by Treatment Group, With Additional Adjustment for Gestational Diabetes eTable 3. Time to Full Enteral Feed, by Treatment Group, Restricted to Eligible Infants eTable 4. Time to Full Enteral Feed, by Treatment Group, Restricted to Infants Born ≥34 Weeks’ Gestational Age eAppendix 2. Protocol Deviations eReference. [file jamapediatr-e252365-s003.pdf]

Supplemental Online Content

Rumbold AR, Lai MM, August D, et al. Supplemental donor milk vs infant formula in moderate to late preterm infants: a randomized clinical trial. *JAMA Pediatr*. Published online August 4, 2025. doi:10.1001/jamapediatrics.2025.2365

eTable 1. Which Group Mothers Thought Their Baby Was In, by Treatment Group

eAppendix 1. Sensitivity Analyses for the Primary Outcome

eTable 2. Time to Full Enteral Feeds, by Treatment Group, With Additional Adjustment for Gestational Diabetes

eTable 3. Time to Full Enteral Feed, by Treatment Group, Restricted to Eligible Infants

eTable 4. Time to Full Enteral Feed, by Treatment Group, Restricted to Infants Born ≥34 Weeks' Gestational Age

eAppendix 2. Protocol Deviations

eReference.

This supplemental material has been provided by the authors to give readers additional information about their work.

© 2025 Rumbold AR et al. *JAMA Pediatrics*.

eTable 1. Which Group Mothers Thought Their Baby Was In, by Treatment Group*

Suspected treatment group	Donor Milk	Formula	Overall
Donor milk group	13/67 (19.4)	17/76 (22.4)	30/143 (21.0)
Formula group	6/67 (9.0)	10/76 (13.2)	16/143 (11.2)
Unsure	48/67 (71.6)	49/76 (64.5)	97/143 (67.8)
Blinding index**	0.104	-0.092	

*Excludes 25 mothers in the donor milk group and 6 mothers in the formula group who did not provide a response.

**The blinding index takes a value between -1 and 1 and represents the proportion of participants who correctly/incorrectly guessed their treatment allocation within each arm (a value > 0 implies more participants guessed correctly, a value < 0 implies more participants guessed incorrectly, and an index of 0 indicates random guessing i.e. successful blinding). A blinding index between -0.2 and 0.2 is generally indicative of successful blinding.[1]

© 2025 Rumbold AR et al. *JAMA Pediatrics*.

eAppendix 1. Sensitivity Analyses for the Primary Outcome

Post-hoc sensitivity analyses that included adjustment for gestational diabetes, exclusion of the 4 infants who did not meet eligibility criteria, and restriction to the ≥34 weeks GA stratum (where both participating sites provided data) did not alter the findings for the primary outcome (eTables 2, 3 and 4).

© 2025 Rumbold AR et al. *JAMA Pediatrics*.

eTable 2. Time to Full Enteral Feeds, by Treatment Group, With Additional Adjustment for Gestational Diabetes*

Outcome	Donor Milk N included	Formula N included	Donor Milk	Formula	Difference in means (95% CI)	P
Time to full enteral feeds, days	90	95	5.6 ± 2.5	5.9 ± 3.5	-0.15 (-1.00, 0.70)	0.73

*Excludes infants of mothers with unknown gestational diabetes status
Treatment effect expressed as difference in means (Donor Milk – Formula).

© 2025 Rumbold AR et al. *JAMA Pediatrics*.

eTable 3. Time to Full Enteral Feed, by Treatment Group, Restricted to Eligible Infants

Outcome	Donor Milk N included	Formula N included	Donor Milk	Formula	Difference in means (95% CI)	p
Time to full enteral feeds, days	96	101	5.6 ± 2.4	5.8 ± 3.4	-0.16 (-0.98, 0.67)	0.71

Treatment effect expressed as difference in means (Donor Milk – Formula)

© 2025 Rumbold AR et al. *JAMA Pediatrics*.

eTable 4. Time to Full Enteral Feed, by Treatment Group, Restricted to Infants Born ≥34 Weeks' Gestational Age

Outcome	Donor Milk<br>N included	Formula<br>N included	Donor Milk	Formula	Difference in means (95% CI)	p
Time to full enteral feeds, days	74	72	5.5 ± 2.2	5.3 ± 2.1	0.22 (-0.48, 0.92)	0.54

Treatment effect expressed as difference in means (Donor Milk – Formula)

© 2025 Rumbold AR et al. *JAMA Pediatrics*.

eAppendix 2. Protocol Deviations

One participant was given formula prior to randomization, thus incorrectly randomized. Following randomization, three participants were diagnosed with a medical condition that affects feeding. All of these infants are included in the main trial analyses.

There were 13 protocol deviations recorded. These included: given non-protocol formula during intervention period (n=7), given study milk after the intervention period ended (n=2), treating health care team accidentally unblinded (n=3), study milk given when there was sufficient maternal milk available (n=1).

© 2025 Rumbold AR et al. *JAMA Pediatrics*.

eReference.

1. Bang H, Ni L, Davis CE. Assessment of blinding in clinical trials. *Control Clin Trials*. 2004;25(2):143-56.

© 2025 Rumbold AR et al. *JAMA Pediatrics*.
