# Supplementary material for: Supplemental Donor Milk vs Infant Formula in Moderate to Late Preterm Infants: A Randomized Clinical Trial
Source: JAMA Pediatr. 2025 Aug 4;179(10):1065–73. doi: 10.1001/jamapediatrics.2025.2365 (PMC12322819; doi:10.1001/jamapediatrics.2025.2365)
Supplement: Supplement 4. — Data Sharing Statement [file jamapediatr-e252365-s004.pdf]

Data Sharing Statement

Rumbold. Supplemental Donor Milk vs Infant Formula in Moderate to Late Preterm Infants. *JAMA Pediatr*. Published August 04, 2025. doi:10.1001/jamapediatrics.2025.2365

Data

Additional Information: Australian and New Zealand Clinical Trial Registry https://www.anzctr.org.au/ Trial registration: ACTRN12621000529842
Data available: Yes
Data types: Deidentified participant data, Data dictionary
How to access data: De-identified data may be made available for sharing upon request to the trial steering committee via alice.rumbold@sahmri.com
When available: With publication

Supporting Documents

Document types: Other (please specify)
Additional Information: Statistical analysis plan and trial protocol
How to access documents: alice.rumbold@sahmri.com
When available: With publication

Additional Information

Who can access the data: Researchers whose proposed use of the data has been approved
Types of analyses: For a specified purpose
Mechanisms of data availability: Signed data access agreement
Any additional restrictions: Nil
